# The Ty1 Retrotransposon Restriction Factor p22 Targets Gag

**DOI:** 10.1371/journal.pgen.1005571

**Published:** 2015-10-09

**Authors:** Jessica M. Tucker, Morgan E. Larango, Lucas P. Wachsmuth, Natarajan Kannan, David J. Garfinkel

**Affiliations:** Department of Biochemistry and Molecular Biology, University of Georgia, Athens, Georgia, United States of America; University of Utah School of Medicine, UNITED STATES

## Abstract

A novel form of copy number control (CNC) helps maintain a low number of Ty1 retrovirus-like transposons in the *Saccharomyces* genome. Ty1 produces an alternative transcript that encodes p22, a *trans*-dominant negative inhibitor of Ty1 retrotransposition whose sequence is identical to the C-terminal half of Gag. The level of p22 increases with copy number and inhibits normal Ty1 virus-like particle (VLP) assembly and maturation through interactions with full length Gag. A forward genetic screen for CNC-resistant (CNC^R^) mutations in Ty1 identified missense mutations in *GAG* that restore retrotransposition in the presence of p22. Some of these mutations map within a predicted UBN2 domain found throughout the Ty1/*copia* family of long terminal repeat retrotransposons, and others cluster within a central region of Gag that is referred to as the CNC^R^ domain. We generated multiple alignments of yeast Ty1-like Gag proteins and found that some Gag proteins, including those of the related Ty2 elements, contain non-Ty1 residues at multiple CNC^R^ sites. Interestingly, the Ty2-917 element is resistant to p22 and does not undergo a Ty1-like form of CNC. Substitutions conferring CNC^R^ map within predicted helices in Ty1 Gag that overlap with conserved sequence in Ty1/*copia*, suggesting that p22 disturbs a central function of the capsid during VLP assembly. When hydrophobic residues within predicted helices in Gag are mutated, Gag level remains unaffected in most cases yet VLP assembly and maturation is abnormal. Gag CNC^R^ mutations do not alter binding to p22 as determined by co-immunoprecipitation analyses, but instead, exclude p22 from Ty1 VLPs. These findings suggest that the CNC^R^ alleles enhance retrotransposition in the presence of p22 by allowing productive Gag-Gag interactions during VLP assembly. Our work also expands the strategies used by retroviruses for developing resistance to Gag-like restriction factors to now include retrotransposons.

## Author Summary

The presence of transposable elements in the eukaryotic genome threatens genomic stability and normal gene function, thus various defense mechanisms exist to silence element expression and target integration to benign locations in the genome. Even though the budding yeast *Saccharomyces* lacks many of the defense systems present in other eukaryotes, including RNAi, DNA methylation, and APOBEC3 proteins, they maintain low numbers of mobile elements in their genome. In the case of the *Saccharomyces* retrotransposon Ty1, a system called copy number control (CNC) helps determine the number of elements in the genome. Recently, we demonstrated that the mechanism of CNC relies on a *trans*-acting protein inhibitor of Ty1 expressed from the element itself. This protein inhibitor, called p22, impacts the replication of Ty1 as its copy number increases. To identify a molecular target of p22, mutagenized Ty1 was subjected to a forward genetic screen for CNC-resistance. Mutations in specific domains of Gag, including the UBN2 Gag motif and a novel region we have named the CNC^R^ domain, confer CNC^R^ by preventing the incorporation of p22 into assembling virus-like particles (VLPs), which restores maturation and completion of the Ty1 life cycle. The mechanism of Ty1 inhibition by p22 is conceptually similar to Gag-like restriction factors in mammals since they inhibit normal particle function. In particular, resistance to p22 and the enJS56A1 restriction factor of sheep involves exclusion of the restriction factor during particle assembly, although Ty1 CNC^R^ achieves this in a way that is distinct from the Jaagsiekte retrovirus escape mutants. Our work introduces an intriguing variation on resistance mechanisms to retroviral restriction factors.

## Introduction

The Ty1 and Ty2 retrotransposons of *Saccharomyces* belong to the Ty1/*copia* group of long terminal repeat (LTR) retrotransposons which replicate in a manner analogous to retroviruses [1]. Ty1 is the most abundant of five retrotransposon families (Ty1-Ty5) in the S288C reference genome of *Saccharomyces cerevisiae*, followed by the related Ty2 element [2, 3]. Recently, Ty2 has been shown to outnumber Ty1 in some *Saccharomyces* genomes [2–4], but Ty1 remains the more widely studied retrotransposon [1]. Ty1 contains two overlapping ORFs, *GAG* and *POL*, and many elements are transpositionally competent and transcriptionally active [5]. An abundant full-length Ty1 mRNA is transcribed which serves as a template for translation and reverse transcription. Two translation products are produced: Gag (p49) and Gag-Pol (p199), of which the latter comprises only 5–10% of total translation products due to its production requiring a +1 ribosomal frameshifting event. Gag, Gag-Pol and Ty1 mRNA accumulate in the cytoplasm in distinct foci called retrosomes [6–9]. Virus-like particles (VLPs) assemble from Gag and Gag-Pol proteins within retrosomes and encapsidate Ty1 mRNA, and tRNA^iMet^, which is used to prime reverse transcription. VLP maturation occurs via the activity of the *POL*-encoded enzyme, protease (PR). Pol is cleaved from p199 via a PR-dependent autocatalytic event, followed by PR cleavage of Gag at its C-terminus (from p49 to p45) and Pol at two internal sites to form mature PR, integrase (IN), and reverse transcriptase (RT). Once maturation occurs, reverse transcription of the packaged genomic Ty1 RNA forms a cDNA copy that is integrated into the host genome. Because Ty1 insertions can mutate cellular genes and mediate chromosome instability by homologous recombination with elements dispersed in the genome, it is beneficial to the host to control the process of retrotransposition [10–13].

Natural isolates of *S*. *cerevisiae* and its closest relative *S*. *paradoxus* maintain lower copy numbers of the Ty1 retrotransposon in their genomes compared to the reference laboratory strain S288C [2–4, 14], without the support of eukaryotic defense mechanisms such as RNAi or the presence of innate restriction factors like the APOBEC3 proteins [15–19]. The maintenance of Ty1 copy number is due at least in part to a mechanism called copy number control (CNC), which was first observed in an isolate of *S*. *paradoxus* that lacks complete Ty1 elements but contains numerous solo-LTRs [20]. The Ty1-less strain supports higher levels of Ty1 transposition compared to standard lab strains, as monitored using a Ty1 tagged with the *his3-AI* retrotransposition indicator gene [21]. Additionally, Ty1*his3-AI* mobility decreases dramatically when the naive genome is repopulated with Ty1 elements [20]. Introduction of a transcriptionally repressed Ty1 element on a multi-copy plasmid also inhibits Ty1*his3-AI* mobility. Based on these observations, CNC is conferred by a factor produced directly by the Ty1 element. The CNC phenotype, which includes decreased levels of transposition [20], the reduction of mature Ty1 RT and PR protein levels, and the absence of detectable mature IN [22], is dependent on the *GAG* open reading frame [20]. Overexpression of Ty1 fused to a *GAL1* promoter on a multi-copy plasmid has been shown to override CNC, suggesting that CNC can be saturated [23–26].

Recently, we found that CNC functions through the protein product encoded by a subgenomic internally-initiated Ty1 sense transcript, called Ty1i (internal) RNA [26]. Transcription of Ty1i RNA initiates within *GAG*, about 800 nucleotides downstream of the full-length, transposition-competent Ty1 mRNA. The first AUGs are in the same reading frame as Ty1 Gag, resulting in synthesis of a 22 kD protein (p22) that shares the same sequence as the C-terminal half of Gag [26, 27]. This shared sequence includes the PR cleavage site, which is utilized within the inhibitory protein to form p18 [26]. Ectopic co-expression of p22 or p18 with Ty1 dramatically inhibits Ty1 mobility. p22/p18 co-immunoprecipitates with Ty1 Gag and co-localizes with Ty1 Gag in the cytoplasm. Ectopic expression of p22/p18 disrupts normal retrosome formation and VLP assembly, followed by a block in maturation and reverse transcription within the VLPs that are able to form. In addition, p18 interferes with the nucleic acid chaperone (NAC) function of Gag, further disrupting Ty1 replication [27, 28]. It is not clear which insult by p22/p18 is most destructive, but collectively these effects result in the strong inhibition of retrotransposition observed during CNC.

Retroelement restriction mechanisms have been aided by studying resistance mutations in retroviruses and/or sequence variation determining viral tropism. An example of particular relevance is the discovery that capsid (CA), a cleavage product of retroviral Gag, is the target of several restriction factors including Friend virus susceptibility factor–1 (Fv1), tripartite motif 5 alpha (TRIM5α), and myxovirus resistance protein 2 (Mx2), among others [29–33]. Viruses that escape restriction by these factors typically carry mutations in the CA-encoding region of the *gag* gene [29, 34–38]. In the case of Fv1 and TRIM5α, viral escape mutations disrupt binding between the restriction factor and CA by altering CA surface residues [37–40], while Mx2 escape mutations in CA are not fully understood but likely alter the interactions between CA and host factors [32, 33, 41]. While Fv1, TRIM5α, and Mx2 bind the incoming viral capsid during the early stages of retroviral infection, the sheep restriction factor enJS56A1 is known to interact with Jaagsiekte sheep retrovirus (JSRV) Gag at later stages when the integrated provirus is undergoing translation and particle assembly [42]. Resistance to enJS56A1 is conferred by mutations in the signal peptide of the JSRV envelope glycoprotein, which is hypothesized to ultimately alter the ratio of JSRV to enJS56A1 Gag levels to favor JSRV particle production [43]. Remarkably, Fv1 and enJS56A1 are both derived from endogenous retroelement *gag* genes [42, 44], similar to p22.

To further understand the mechanism of CNC, we carried out forward genetic screens for CNC-resistant (CNC^R^) Ty1 elements. Almost all of the CNC^R^ elements contain missense mutations within *GAG* that map within predicted helices important for VLP assembly and maturation. Computational and functional analyses reveal three domains within the Ty1 Gag protein: TYA, CNC^R^ and UBN2. All resistance mutations recovered map within the CNC^R^ and UBN2 domains encoded by *GAG*. Importantly, several mutations are not present in p22 coding sequence, supporting the idea that p22 targets Gag to inhibit retrotransposition. Most CNC^R^ mutations in *GAG* do not markedly alter Ty1 fitness or the interaction between Gag and p22, but prevent co-assembly of Gag and p22 into VLPs, which improves VLP maturation and progression through the retrotransposon life cycle.

## Results

### Copy number control in an *S*. *paradoxus spt3Δ* mutant

**Fig 1:**
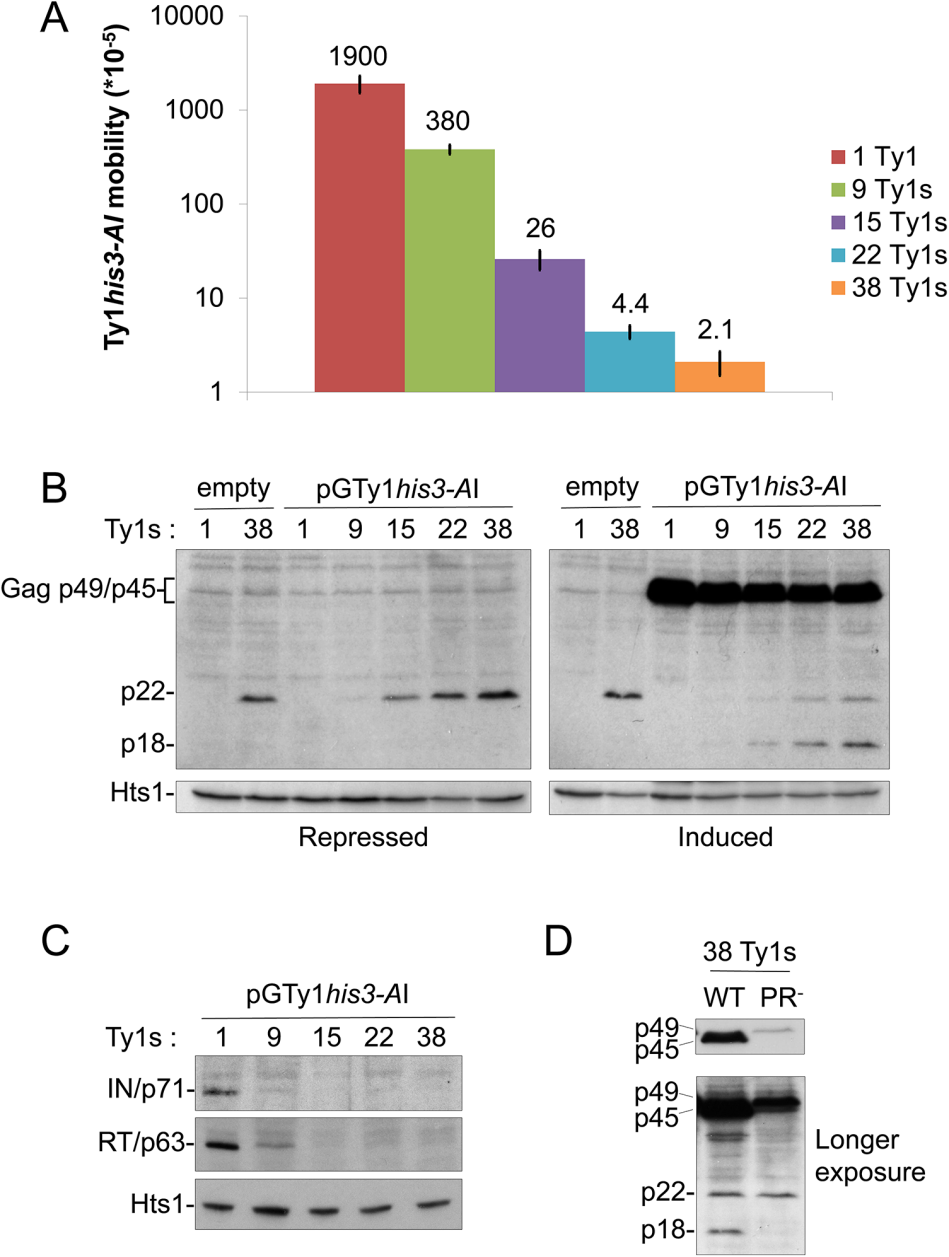
Ty1 mobility and protein levels in cells repopulated with Ty1 elements. A Ty1-less *S*. *paradoxus* strain repopulated with increasing copies of genomic Ty1-H3 elements [YEM514 (1 Ty1), YEM568 (9 Ty1s), YEM570 (15 Ty1s), YEM572 (22 Ty1s), and YEM515 (38 Ty1s)] and containing pGTy1*his3-AI*/*URA3*/*CEN* (pBDG606) were analyzed for Ty1*his3-AI* mobility and protein levels. All strains were deleted for *SPT3*, which blocks Ty1 mRNA, but not Ty1i RNA, expression from genomic loci. (A) A quantitative Ty1*his3-AI* mobility assay was performed with galactose-induced cells. Numbers represent Ty1*his3-AI* mobility events per cell and bars represent standard deviations. Mobility assays were repeated at least three times and representative results are shown. (B) Cells were grown for 24 h under repressing (glucose) or inducing (galactose) conditions for pGTy1*his3-AI* expression. 1 and 38 Ty1 copy strains containing an empty vector were used as controls. TCA-precipitated protein extracts were immunoblotted with p18 antibody, which recognizes full length immature and mature Gag (p49/p45) and p22/p18, and Hts1 antibody, which served as a loading control. Separation of Gag-p49 and p45 does not occur under these electrophoresis conditions (see Materials and Methods) and thus are bracketed and collectively labeled as Gag-p49/p45. (C) Cells were induced for pGTy1 expression for 24 h and protein extracts were immunoblotted with IN, RT, or Hts1 antibody. (D) The 38 Ty1 copy strain containing WT or PR-defective (SacI linker–1702 [47]) pGTy1/2*μ* plasmids were induced with galactose for 24 h. TCA-precipitated extracts were immunoblotted with p18 antibody.

We hypothesized that the generation of CNC^R^ Ty1 mutants may identify a molecular target of p22. Since previous work implicated a physical interaction between Gag and p22 [26], isolating resistance mutations in *GAG* would suggest that this interaction is important for CNC. To identify Ty1 mutants that are resistant to the effects of p22 and its processed form p18, we designed a system that allowed for simultaneous expression of wild type p22/p18 and a randomly mutagenized Ty1*his3-AI* element fused to the regulated *GAL1* promoter carried on a low copy centromere-based (*CEN*) plasmid (pGTy1*his3-AI*/*CEN*). The purpose of using a low copy plasmid for pGTy1*his3-AI* expression was to minimize CNC saturation that occurs with overexpression of Ty1 on a multi-copy plasmid. In addition, the Ty1 copy number provided by a low copy centromere-based plasmid does not result in detectable CNC [22]. Isogenic, repopulated Ty1-less *S*. *paradoxus* strains containing 1–38 copies of Ty1-H3 were analyzed, representing a wide range of Ty1 copy numbers naturally found in yeast (S1 Table) [2, 3, 14]. All strains carried a deletion of *SPT3*, which encodes a transcription factor required for expression of full length Ty1 mRNA from nucleotide 238 (Ty1-H3, Genbank M18706.1) and the synthesis of Ty1 Gag and Gag-Pol [45]. Ty1i RNA, which initiates internally at nucleotide ~1000, is still produced in *spt3Δ* mutants [26, 45, 46]. pGTy1*his3-AI*/*CEN* provided Ty1 mRNA, Gag and Gag-Pol and the strains were analyzed for CNC (Fig 1). Ty1 mRNA produced from this plasmid contains the *his3-AI* indicator gene, allowing transposition levels to be analyzed by growth on media lacking histidine. As expected, increasing Ty1 copy number resulted in decreased Ty1 mobility, with the strongest decrease observed in the presence of 38 genomic copies of Ty1 (Fig 1A). These strains were immunoblotted for p22/p18 levels in the presence and absence of pGTy1*his3-AI* expression using p18 antiserum, which detects both Gag-p49/p45 and p22/p18 [26]. Under both repressing (glucose) and inducing (galactose) growth conditions, p22 levels in cell extracts increased similarly with copy number (Fig 1B). Because p22 was not detected in the lowest Ty1 copy strain (1 Ty1) containing pGTy1*his3-AI*, these results confirmed that pGTy1*his3-AI* does not produce detectable p22. It remains possible that increasing chromosomal copies of Ty1 stimulated p22 production from pGTy1*his3-AI*, but this seems unlikely considering that p22 levels do not increase in the 38 Ty1 copy strain containing pGTy1*his3-AI* compared to an empty vector in either growth condition (Fig 1B). Therefore, genomic Ty1-H3 elements, and not the pGTy1*his3-AI* mutant library, were the source of p22 in the screen. When pGTy1*his3-AI* expression was induced by galactose, Gag-p49/p45 was detected and the maturation of p22 to p18 was observed, supporting previous findings suggestive of p22 cleavage by Ty1 PR [26]. In addition, mature RT (p63) and IN (p71) were present only in low copy number strains (Fig 1C), confirming another feature of the CNC phenotype [22]. To further establish that cleavage of p22 was Ty1 PR-dependent, wild type or PR-defective pGTy1/*2μ* multi-copy plasmids were introduced into the 38 Ty1 copy strain (Fig 1D). As expected, neither Gag-p49 expressed from the PR^-^ Ty1 nor p22 expressed from genomic elements were cleaved to form mature products Gag-p45 and p18, respectively (Fig 1D).

### CNC^R^ mutations are in *GAG*

To search for pGTy1*his3-AI* CNC^R^ mutations, we utilized the 38 Ty1 copy strain described above, which produced the highest level of p22 of the strains tested (Fig 1B). pGTy1*his3-AI* was mutagenized by propagation in a mutator strain of *E*. *coli* (XL–1 Red, Agilent Technologies, Santa Clara, CA) and 20,000 transformants were screened for an increase in Ty1*HIS3* mobility following induction on medium containing galactose (see Materials and Methods *)*. The CNC region (nucleotides 238–1702 [48]) was sequenced from pGTy1*his3-AI* plasmids that conferred an increase in Ty1 mobility when compared to wild type plasmid controls. Nine unique mutations were present in *GAG* (S2 Table; XL–1 Red). A restriction fragment encompassing the CNC region was subcloned from the mutant plasmids into a fresh pGTy1*his3-AI* vector to eliminate contribution of background mutations and no loss of CNC^R^ was observed. To avoid bias based on our mutagenesis method and to generate additional CNC^R^ mutations, *GAG* and *POL* were mutagenized separately by error-prone PCR, followed by gap-repair with pGTy1*his3-AI* in the 38 Ty1 copy strain. While *GAG* mutagenesis by PCR revealed 8 new CNC^R^ mutations from 500 colonies, *POL* mutagenesis revealed only 1 CNC^R^ candidate from 6,000 colonies (S2 Table). While most of the mutations (8 of 9) isolated via XL–1 Red mutagenesis were single base changes, 5 of 8 mutations recovered via PCR mutagenesis had more than one base change. Interestingly, all *GAG* mutations recovered with either method were missense, suggesting that they function at the level of the Gag protein. The only CNC^R^ pGTy1*his3-AI* plasmid isolated from *POL* mutagenesis contained two missense mutations within RT (D518G/V519A).

**Fig 2:**
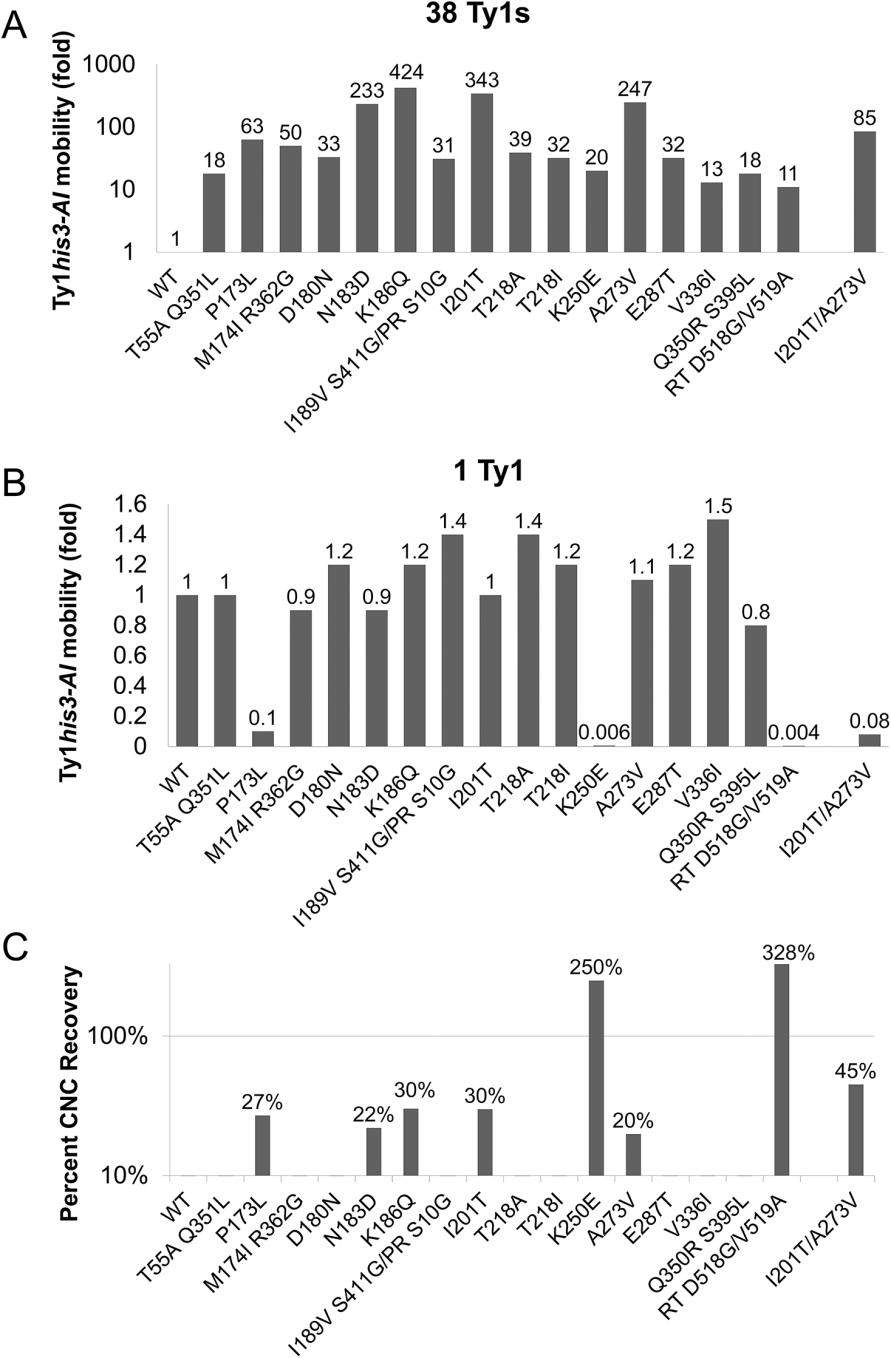
Ty1 *GAG* mutations are specific and partially resistant to CNC. *S*. *paradoxus* repopulated with 38 (YEM515; A) or 1 (YEM514; B) genomic copies of Ty1-H3 and containing wild type or CNC^R^ pGTy1*his3-AI* plasmids were analyzed for Ty1*his3-AI* mobility. Ty1*his3-AI* mobility for each mutant was normalized to wild type and fold changes are represented. Raw data can be found in S4 Table. (C) Percent CNC recovery was calculated by dividing Ty1*his3-AI* mobility in the 38 Ty1 copy strain by mobility in the 1 copy strain. Recoveries above 10% are shown.

To quantify the level of resistance to p22/p18, the frequency of Ty1*his3-AI* mobility was determined for the mutants alongside wild type controls (Fig 2, S4 Table). In the 38 Ty1 copy strain, most mutant plasmids produced mobility frequencies between 11- and 63- fold higher than wild type (Fig 2A). Four candidates from the CNC^R^ screen (Gag N183D, K186Q, I201T, and A273V) exhibited stronger resistance, ranging from 233- to 424-fold higher than wild type (Fig 2A). Because it was possible to obtain Ty1 mutations that globally increased transposition, rather than acting specifically in the presence of p22/p18, CNC^R^ mobility was also measured in the 1 Ty1 copy strain (Fig 2B). Importantly, all CNC^R^ mutants transposed at similar or decreased levels compared to wild type in the absence of p22/p18, indicating that we obtained CNC-dependent mutations. Three CNC^R^ mutants, Gag P173L, Gag K250E, and RT D518G/V519A, showed a decrease in Ty1*his3-AI* mobility in the absence of p22/p18, indicating that these mutations negatively impacted Ty1 fitness (Fig 2B). Percent recovery of Ty1*his3-AI* mobility with CNC^R^ plasmids was calculated by dividing Ty1*his3-AI* mobility in the presence or absence of p22 (Percent CNC Recovery, Fig 2C). As expected, the three CNC^R^ mutations with decreased fitness showed higher percent recovery, with K250E and RT D518G/V519A at >100%, due to the fact that these plasmids result in higher transposition frequencies in the presence of p22 than in its absence. Since overall Ty1*his3-AI* mobility was extremely low, further studies were not performed with these mutants. The remaining four mutations conferring >10% CNC^R^ include those resulting in Gag amino acid changes N183D, K186Q, I201T, and A273V. These elements exhibited 20–30% recovery, indicating that while the strongest CNC^R^ mutations dramatically increase transposition in the presence of p22 (Fig 2A), they are only partially resistant to the effects of p22. Note that both classes of recovery should be expected since the mutant screen balanced transposition fitness and CNC^R^. Consequently, we focused on the N183D, K186Q, I201T, and A273V *GAG* mutations, since they conferred the strongest levels of resistance recovered with no effect on fitness of Ty1.

In an effort to increase CNC^R^, cells containing double mutant pGTy1*his3-AI-K186Q/I201T* or pGTy1*his3-AI-A273V/I201T* were tested for Ty1*his3-AI* mobility. Gag K186Q/I201T was defective for transposition and was not studied further. Gag A273V/I201T was able to transpose, but experienced decreased fitness. In the 38 Ty1 copy strain, the levels of Ty1*his3-AI* mobility with A273V/I201T was lower than either single mutant, but still 85-fold higher than wild type (Fig 2A). In the 1 Ty1 copy strain, A273V/I201T exhibited Ty1*his3-AI* mobility at 8% of wild type levels (Fig 2B). Interestingly, the double mutant did exhibit increased CNC recovery (45%), but at the expense of overall Ty1 mobility (Fig 2C). The loss of fitness in the double mutants reinforces the idea that mutations conferring a high level of CNC^R^ may have been missed in the screen since they compromise Ty1 fitness. In addition, Ty1 Gag may be genetically fragile since it cannot tolerate wholesale alterations in sequence, a feature that is also observed with HIV–1 CA [49].

### CNC^R^ mutations N183D, K186Q, I201T, and A273V function in a genomic context

To determine if the CNC^R^ elements are resistant to p22/p18 in a genomic context, wild type or CNC^R^ pGTy1*his3-AI*/*CEN* plasmids were expressed in Ty1-less *S*. *paradoxus*, and cells with 1–2 wild type or CNC^R^ Ty1*his3-AI* genomic insertions were identified. Next, an empty vector or a p22-producing plasmid, pTy1-ATGfs (S3 Table), was introduced into these strains. In the absence of p22 (Table 1, empty vector), genomic CNC^R^ Ty1 elements N183D, K186Q, and I201T transposed at similar levels to the respective wild type control (<2-fold change), confirming that they do not globally increase Ty1*his3-AI* mobility in a chromosomal context. In contrast, CNC^R^ Ty1 A273V displayed a 4.3-fold increase in Ty1 mobility compared to wild type in the presence of empty vector, indicating that A273V may not be CNC-dependent in all contexts. This may be due to the fact that A273V is the only mutation tested that maps within *GAG* and p22 coding sequence, thus changes in both proteins could be affecting Ty1 mobility. Dramatic differences in CNC were observed when the wild type and CNC^R^ Ty1*his3-AI* elements are challenged with p22 (Table 1, pTy1-ATGfs). While p22 expression decreases wild type Ty1*his3-AI* mobility 56- to 120- fold, CNC^R^ Ty1*his3-AI* mobility is partially resistant to the effects of p22, decreasing 2- to 13-fold.

**Table 1:** Genomic CNC-resistant Ty1*his3-AI* mobility.

Strain	# Ty1*his3-AI*	empty vector (-p22) X 10^−6^ (SD)	pTy1-ATGfs (+p22) X 10^−6^ (SD)	Fold decrease in Ty1*his3-AI* mobility
DG3710	2 WT	95 (12)	1.7 (0.3)	56
DG3716	2 N183D	160 (20)	37 (15)	4.3
DG3713	1 WT	110 (14)	1.5 (0.3)	73
DG3725	1 K186Q	83 (29)	22 (8.3)	4
DG3713	1 WT	38 (12)	0.32 (0.14)	120
DG3722	1 I201T	37 (7.0)	19 (4.0)	2
DG3713	1 WT	23 (6.0)	0.24 (0.04)	98
DG3719	1 A273V	100 (14)	7.6 (1.3)	13

A key feature of CNC is a decrease in Ty1 mobility of a single genomic Ty1*his3-AI* in the presence of elevated Ty1 copies [48]. In contrast, additional chromosomal copies of CNC-defective Ty1 elements, which are elements that do not produce p22/p18 but retain the ability to undergo retrotransposition, increase the level of Ty1*his3-AI* mobility [26]. To determine how chromosomal CNC^R^ elements influence Ty1*his3-AI* mobility (Table 2), *S*. *paradoxus* containing a wild type Ty1*his3-AI* genomic insertion was repopulated with unmarked CNC^R^ elements carrying the N183D, K186Q, I201T, and A273V mutations. It is important to note that Ty1*his3-AI* RNA is not preferentially packaged *in cis* [23] and can serve as the genomic RNA in mixed particles containing wild type and CNC^R^ Gag, with the latter likely being produced in excess due to increased genomic copy number. Compared with the starting strain, Ty1*his3-AI* mobility in strains repopulated with +14 and +21 wild type Ty1 elements decreased 31- and 620-fold, respectively. Repopulation with +14–20 CNC^R^ elements did not alter Ty1*his3-AI* mobility, supporting the idea that CNC^R^ mutations relieve the inhibitory effects of p22 produced by the additional chromosomal elements. However, the fact that additional CNC^R^ elements do not stimulate Ty1*his3-AI* mobility probably reflects the partial resistance phenotype imparted by the CNC^R^ mutations.

**Table 2:** Ty1*his3-AI* mobility in CNC-resistant Ty1 repopulated strains.

Strain	# Ty1	x 10^−5^ (SD)	Fold change in Ty1*his3-AI* mobility
DG2533	0	3.1 (0.9)	1
YEM13	+14 WT	0.1 (0.03)	↓31
YEM14	+21 WT	0.005 (0.002)	↓620
JM487	+18 K186Q	3.5 (0.7)	↑1.1
JM506	+20 K186Q	2.7 (0.4)	↓1.1
JM484	+14 I201T	2.8 (0.8)	↓1.1
JM503	+19 I201T	4.9 (1.8)	↑1.6

### CNC^R^ residues map within predicted helical domains of Gag

**Fig 3:**
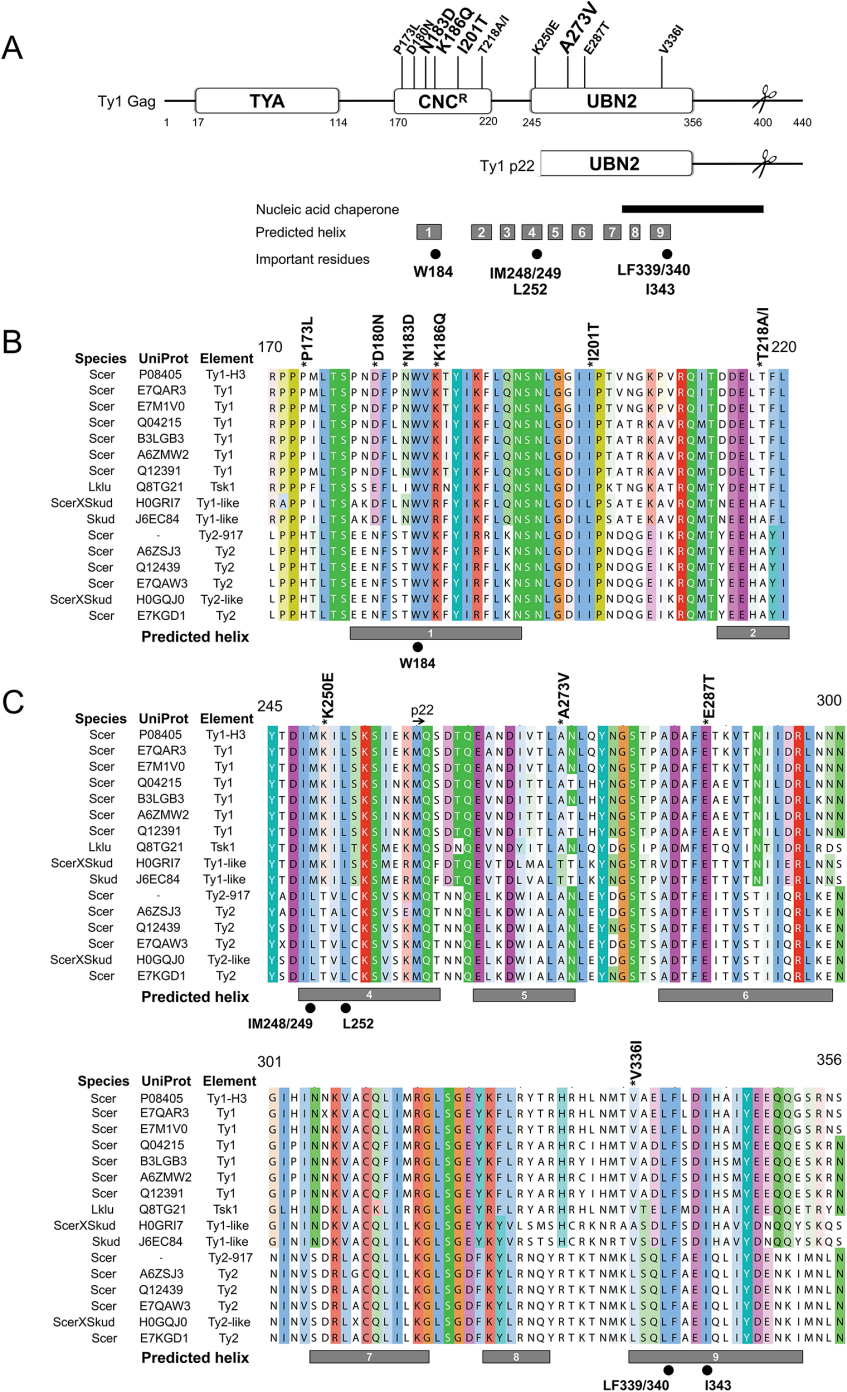
Predicted helical structure of Ty1 Gag and location of CNC^R^ substitutions. (A) Ty1 Gag sequence (UniProt P08405) was analyzed for secondary structure prediction and domain identification. Predicted helical regions are represented by gray boxes below and labeled 1–9. Two Pfam domains, TYA (PF01021, residues 17–114) and UBN2 (PF14223, residues 245–356), are present in Ty1 Gag. Several CNC^R^ residues clustered between residues 170–220, which we define as the CNC^R^ domain. Other features include the Ty1 p22 protein (mapped underneath Ty1 Gag), the PR cleavage sites in Ty1 Gag and p22 proteins (H402/N403 in Gag, scissors) [54], a region that exhibits nucleic acid chaperone (NAC) activity (black bar) [28], and important Gag amino acids (black circles): a highly conserved tryptophan residue in Ty1/copia (W184) [53], and hydrophobic residues previously shown to be important for assembly (IM248/249, L252, LF339/340, and I343) [55]. (B, C) An alignment of yeast Ty1-like Gag sequences was generated with ClustalW and visualized with Jalview using the ClustalX color scheme. The CNC^R^ (B) and UBN2 (C) domains were chosen for display. Species, UniProt annotations, and element type are listed to the left and include Ty1 and Ty2 elements from *S*. *cerevisiae* (Scer), Ty1-like elements from *S*. *kudriavzevii* (Skud) [3, 56] and the Tsk1 element from *Lachancea kluyveri* (Lklu) [57]. Ty1-H3 and Ty2-917 were included, however, these elements were isolated as spontaneous retrotransposition events and are unique from known genomic elements [58, 59]. Ty1 Gag residue coordinates and CNC^R^ substitutions (above), predicted helical regions (gray boxes, below) and important residues (below) from (A) are labeled.

Since little is known about the structure of Ty1 or other LTR-retrotransposon Gag proteins, we submitted Ty1 Gag protein sequence for secondary structure prediction using I-TASSER [50–52], and several other structural prediction servers (see Materials and Methods). These analyses predicted that a central portion of Ty1 Gag contains nine helical regions (Fig 3A, gray boxes), which overlap previously identified conserved Gag domains A, B and C of Ty1/*copia* family of retrotransposons [53]. Using profile-based methods, we identified two annotated protein families (Pfam) within Ty1 Gag called TYA (TYA transposon protein, PF01021) and UBN2 (gag-polypeptide of LTR copia-type, PF14223) (Fig 3A). The TYA domain is found strictly in yeast and corresponds to an unstructured region in the N-terminal half of Gag between residues 17–114. The UBN2 domain maps to the C-terminal half of Gag between residues 245–356, roughly overlapping Ty1/*copia* conserved Gag domains B and C [53], and is represented across multiple plant and fungal species. Of the 11 single *GAG* mutations that impart resistance to p22, 9 mapped within the helical domains, with 4 mapping within the UBN2 domain. Mutations outside of the UBN2 domain clustered between amino acids 170–220, which we refer to here as the CNC^R^ domain (Fig 3A). The CNC^R^ domain contains sequences belonging to Ty1/*copia* Gag conserved domain A [53], which is characterized by an invariant tryptophan residue corresponding to Ty1 Gag W184. Interestingly, CNC^R^ alleles lie in close proximity to the W184 codon.

Ty1-H3 Gag (Uniprot P08405) sequence was used in a profile hidden Markov model search to identify closely related Gag proteins and an alignment was generated to highlight variations in amino acid sequence in CNC^R^ (Fig 3B) and UBN2 (Fig 3C) domains (refer to S1 Fig for a full alignment). Redundant and partial Gag sequences were purged and the Gag sequence of the Ty2-917 element (GenBank KT203716), which was isolated as a spontaneous *HIS4* mutation [58], was added to the hits. In total, we generated a multiple alignment of 15 sequences representing Ty1 and Ty1-like Gag proteins from 9 different yeast strains in the *Saccharomycetaceae* family [60, 61]. While substitutions of CNC^R^ residues do not naturally occur in known Ty1 Gag sequences from *S*. *cerevisiae*, substitutions in all but one of the CNC^R^ residues (E287) are found in the alignment of non-Ty1 Gag proteins, including those from Ty2 elements, the second most abundant Ty1/*copia* retrotransposon found in the *S*. *cerevisiae* S288C genome [2, 3], and Ty1-like elements present in *Saccharomyces kudriavzevii* and *Lachancea kluyveri* (Fig 3B and 3C). Most substitutions are different than those recovered in our CNC^R^ screen, with the exception of D180N and T218A (Fig 3B). Considering all 10 CNC^R^ residues altered in our screen, 6 of these are not conserved from Ty1 to Ty2.

### Ty2 is not influenced by Ty1 or Ty1-like CNC

To determine if Ty2 undergoes CNC, we analyzed the retrotransposition-competent Ty2-917 element [62]. Unlike pGTy1-H3, which confers CNC when *GAL1*-promoted Ty1 mRNA transcription is repressed, a multi-copy pGTy2-917 plasmid does not inhibit Ty1*his3-AI* mobility [48]. This result suggests that a p22-like protein is either not produced by Ty2-917 or does not affect Ty1 movement. A transcriptionally silent pGTy2-917 also did not affect Ty2-917*his3-AI* mobility, demonstrating that Ty2-917 is not under a Ty1-like form of CNC (S5 Table). In fact, a transcriptionally active Ty2-917 carried on a multi-copy plasmid stimulated Ty2-917*his3-AI* mobility 1.5-fold. Whether all Ty2 elements respond the same way as Ty2-917 will require further investigation. Considering the relationship between Ty1 CNC^R^ mutations and Ty2 residues in the CNC^R^ domain (Fig 3B), we introduced an empty vector or the p22 expression plasmid into a strain containing Ty2-917*his3-AI* to determine if Ty2 mobility was sensitive to inhibition by p22 (S5 Table) [26]. A decrease in Ty2-917 mobility of less than 2-fold was observed in cells expressing Ty1-p22, supporting the idea that Ty2-917 is not sensitive to Ty1 CNC.

### Substitutions at hydrophobic residues in Gag helices disturb Ty1 protein maturation

**Fig 4:**
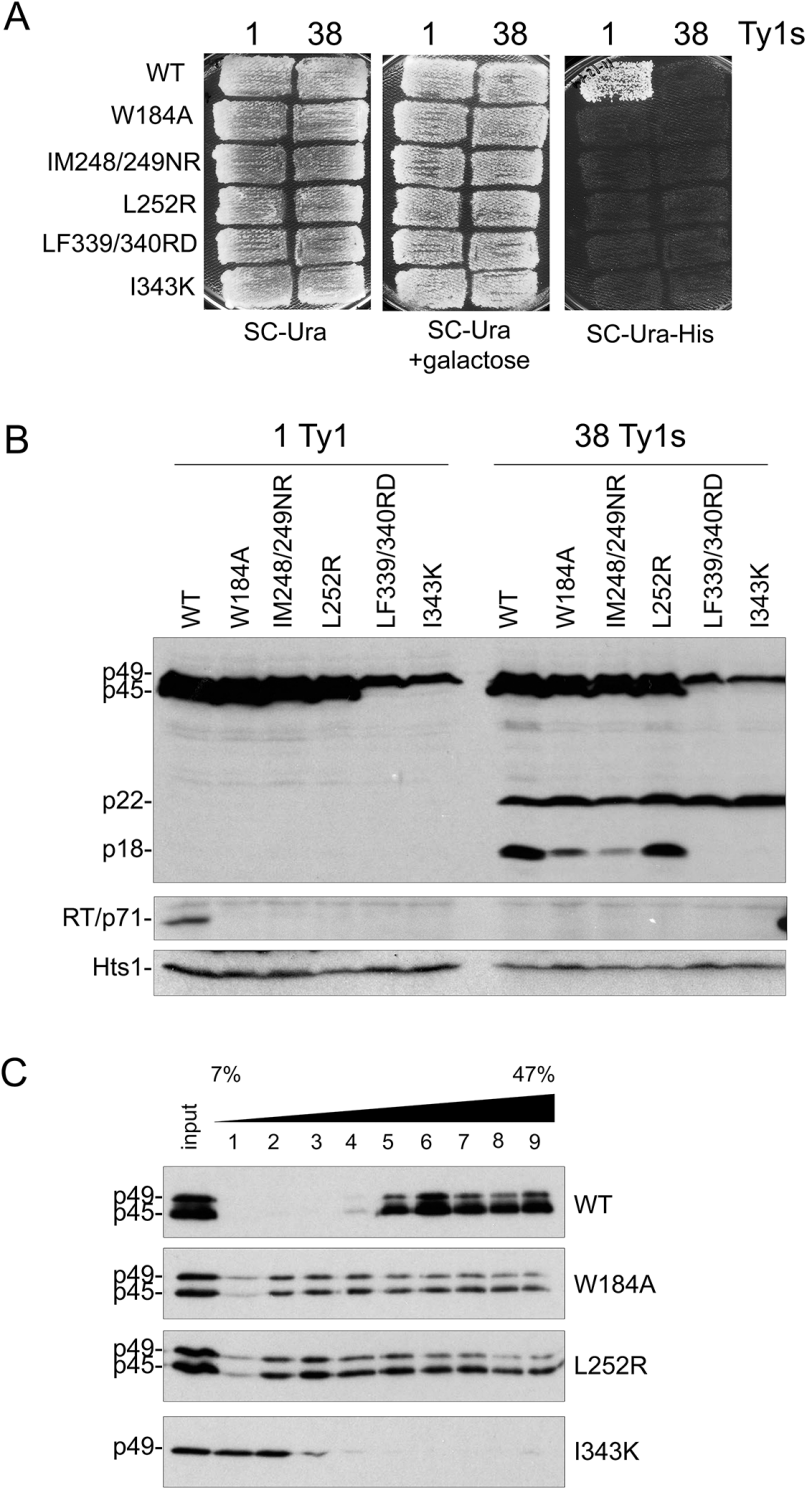
Alterations in Gag helical domains disrupt transposition and Ty1 protein cleavage. *S*. *paradoxus* repopulated with 1 (YEM514) or 38 (YEM515) genomic copies of Ty1-H3 and containing wild type or helix mutant pGTy1*his3-AI* plasmids were analyzed for Ty1*his3-AI* mobility using a qualitative plate assay (A) and for Ty1 protein levels (B). (A) Cells grown on SC-Ura were induced for pGTy1*his3-AI* expression and retrotransposition by replica plating to SC-Ura + 2% galactose and incubating at 22°C for 2 days. Cell patches were replicated to SC-Ura-His and grown at 30°C until His^+^ papillae appeared. Each His^+^ colony contains at least one Ty1*HIS3* insertion. (B) Cells were induced in liquid SC-Ura + 2% galactose medium for 24 h and TCA-extracted proteins were immunoblotted with p18, RT, and Hts1 antibodies. (C) Cell extracts from 1 Ty1 copy strain expressing helix mutant pGTy1*his3-AI* plasmids were centrifuged through a 7–47% continuous sucrose gradient. Equal volumes of each fraction were immunoblotted with TY antibody, which recognizes full length Gag (p49/p45).

Since CNC^R^ residues map to putative helical domains in Gag, a series of mutations were made in hydrophobic residues within several predicted helices (Fig 3A) and their impact on Ty1 transposition and protein levels was analyzed when the mutant elements were expressed from the *GAL1* promoter (Fig 4). We substituted the invariant tryptophan residue found in Ty1/copia Gag proteins to alanine (W184A, helix 1) and tested several published Gag mutations designed to interrupt hydrophobic faces of Gag helices (IM248/249NR, L252R, both in helix 4; LF339/340RD, I343K, both in helix 9) [55]. All helix substitutions abolished Ty1*his3-AI* mobility when expressed in both 1 and 38 Ty1 copy strains (-p22 and +p22, respectively; Fig 4A). Mature RT was not detected in whole cell extracts from the 1 or 38 Ty1 copy strains expressing mutant Ty1, indicating that Gag-Pol maturation did not occur (Fig 4B). When helix 1 (W184A) or helix 4 (IM248/249NR and L252R) was perturbed, Gag was stable and present in both immature (p49) and mature forms (p45), while p22 maturation to p18 was similar or slightly decreased. In contrast, Gag-p49 and p22 from helix 9 substitutions (LF339/340RD and I343K) did not undergo maturation.

We analyzed Gag W184A (helix 1), L252R (helix 4) and I343 K (helix 9) for VLP assembly by sedimentation of total protein extracts through 7–47% sucrose gradients (Fig 4C). This analysis was performed in the 1 Ty1 copy strain to prevent further disturbance by p22 during VLP formation. Wild type Gag migrated primarily as larger complexes, which are probably comprised of assembled VLPs (Fig 4C, fractions 5–9). W184A and L252R VLP assembly was perturbed, as Gag was present in every fraction of the sucrose gradient. There was less cleavage of Gag-p49 to p45 compared to wild type and both forms were present in each fraction. More cleavage of Gag-p49 to p45 was visible in the higher percent sucrose fractions, suggestive of Ty1 PR activity in these fractions. Gag I343K, which did not exhibit Gag cleavage (Fig 4B), remained at the top of the sucrose gradient and did not form higher order structures (Fig 4C).

### CNC^R^ mutations alter Ty1 protein maturation

**Fig 5:**
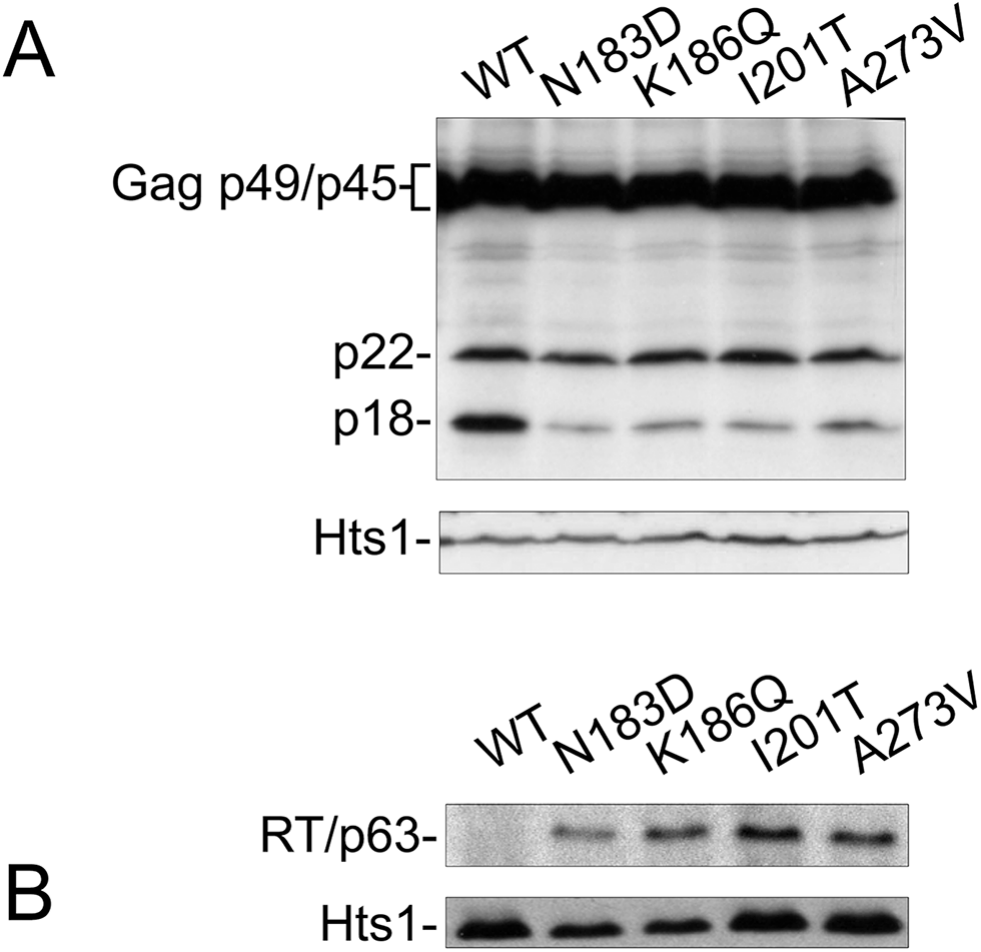
CNC^R^ mutations N183D, K186Q, I201T, and A273V alter Ty1 protein levels. *S*. *paradoxus* repopulated with 38 genomic copies of Ty1-H3 (YEM515) containing wild type or CNC^R^ pGTy1*his3-AI* plasmids were analyzed for Ty1 protein levels. Cells were induced in liquid SC-Ura + 2% galactose for 24 h. (A) TCA-extracted proteins were immunoblotted with p18 and Hts1 antibodies. (B) Whole cell extracts were immunoblotted with RT and Hts1 antibodies.

CNC is associated with altered abundance and maturation of Ty1 proteins, including loss of mature RT and IN [20, 22]. Disturbing Gag helices in which CNC^R^ mutations mapped also affected Ty1 protein maturation (Fig 4). Therefore, Ty1 protein levels produced by CNC^R^ pGTy1*his3-AI* elements were examined. Cell extracts were immunoblotted for Gag-p49/p45 and p22 using the 38 Ty1 copy strain (Fig 5A). When wild type pGTy1*his3-AI* was expressed, there was slightly more p18 than p22. Strikingly, expressing the four CNC^R^ pGTy1*his3-AI* plasmids resulted in a lower level of p18, indicating less cleavage of p22 and/or decreased stability of p18. Mature RT levels were also recovered in the CNC^R^ strains, suggesting that VLP maturation improved (Fig 5B).

**Fig 6:**
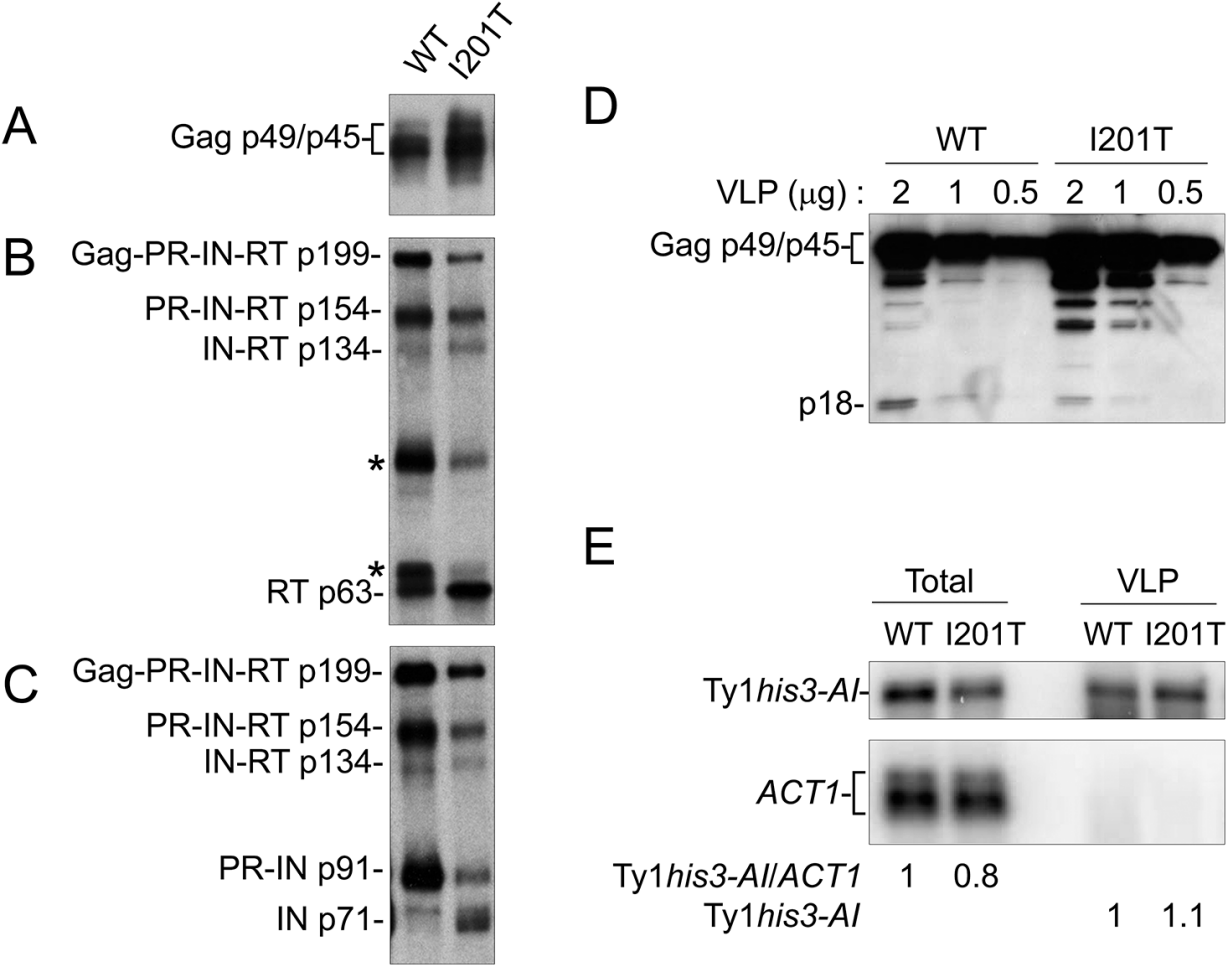
I201T CNC^R^ VLPs have increased IN and RT protein levels and less p18. VLPs were isolated from *S*. *paradoxus* repopulated with 38 genomic copies of Ty1-H3 (YEM515) containing wild type or CNC^R^ pGTy1*his3-AI-I201T* that were induced for expression. Equal amounts (2 μg) of VLPs were immunoblotted with (A) VLP, (B) RT, and (C) IN antibodies. (D) Dilutions of WT and I201T VLPs were analyzed with p18 antibody. (E) Northern blotting of total RNA and VLP RNA was performed using Ty1 (nucleotides 335–550) and *ACT1* ^32^P-labeled riboprobes. Note that *ACT1* transcripts are not detected in purified VLPs.

Ty1 VLPs were isolated from the 38 Ty1 copy strain expressing wild type pGTy1*his3-AI* or pGTy1*his3-AI-I201T* to determine levels of Ty1 protein and RNA within assembled particles (Fig 6). Equal amounts of VLP preparations were immunoblotted for the detection of Gag, RT and IN. Gag protein levels were similar between wild type and I201T VLPs (Fig 6A). Since wild type VLPs represent different stages of maturation, the samples contained Ty1 precursors Gag-PR-IN-RT (Gag-Pol; p199), PR-IN-RT (p154), IN-RT (p134), and PR-IN (p91). In addition, RT antibodies reacted with two bands of unknown origin around 65 and 90 kD, which we reported previously (Fig 6B, asterisks) [26]. As expected from previous work [22], mature IN (p71) was not detected from wild type VLPs isolated from the 38 Ty1 copy strain (Fig 6C). I201T VLP maturation occurred more efficiently, as indicated by increases in mature IN (Fig 6C) and a decrease in the unknown RT-reactive proteins in I201T VLPs (Fig 6B, asterisks). To determine if the increase in Ty1 mature protein products was due to less p22/p18 present in VLPs, dilutions of wild type and I201T VLPs were immunoblotted with p18 antiserum (Fig 6D). p18, rather than p22, was the main protein observed in wild type VLPs, likely due to cleavage by Ty1 PR within VLPs [26]. The level of p18 within I201T VLPs was lower than that observed in wild type VLPs, raising the possibility that less p18 within assembled CNC^R^ VLPs results in increased Ty1 protein maturation or stability.

Ty1 RNA packaging, as demonstrated by protection from digestion when whole cell extracts are treated with the nuclease benzonase [63], is markedly decreased in the presence of p22/p18 [27]. To determine if a CNC^R^ mutation functions by increasing the level of RNA packaged into VLPs, RNA extracted from purified WT and I201T VLPs was subjected to Northern blotting (Fig 6E). Total cellular RNA was examined to control for Ty1 mRNA expression. Wild type and I201T RNA extracts from cells or purified VLPs contained similar levels of Ty1*his3-AI* transcript, suggesting that CNC^R^ does not function through the enhancement of Ty1 RNA packaging, at least in the case of I201T.

Interestingly, p22/p18 shares sequence with two regions implicated in Ty1 nucleic acid transactions: the NAC region of Gag (amino acid residues 299–401) and the N-terminus of PR (known as p4 in Gag) that participates in reverse transcription [27, 28, 64]. The first region was extensively examined using recombinant mature p18, which lacks p4 sequence [27]. Recombinant p18 possesses NAC activity and can bind Ty1 RNA, but truncated versions that lack NAC activity still inhibit Ty1 retrotransposition, suggesting that NAC activity is dispensable for p22/p18 function [27]. To test whether PR/p4 is implicated in CNC, we measured the mobility of a single genomic Ty1*his3-AI* element in presence of transcriptionally repressed wild type pGPOLΔ Ty1 plasmids [26], or derivatives carrying altered p4 regions. Wild type pGPOLΔ plasmids reduced Ty1*his3-AI* mobility by 150-fold compared to an empty *GAL1* plasmid (S6 Table). The pGPOLΔ*d1* plasmid, which carries a deletion in PR/p4 that blocks successful reverse transcription [64, 65], and pYES2-p45 lacking p4 [27] reduced Ty1*his3-AI* mobility by 140- and 160-fold, respectively. These results are supported by the observations that ectopic expression of mature p18 alone, which does not contain PR/p4 sequence, inhibits pGTy1*his3-AI* mobility, and expression of p22/p18 or p22 mutant for the PR cleavage site exhibit similar levels of inhibition [26, 27]. Together, our results show that the PR/p4 region is not required for CNC.

To determine if the low level of p18 detected in I201T VLPs was related to altered binding of p22/p18 with wild type versus Gag I201T, pGp22-V5 and wild type pGTy1*his3-AI* or pGTy1*his3-AI-I201T* were co-expressed in a Ty1-less strain. Endogenous Gag produced by chromosomal Ty1 elements and GST-p18 have been shown to interact via a GST pull-down assay [26]. Functional p22-V5, which carries an internal V5 tag that is present in both p22 and p18, was expressed from a low copy *CEN* plasmid to maximize CNC^R^ imparted by the Gag mutations. Quantitative mobility assays revealed that wild type Ty1*his3-AI* mobility decreased 783-fold in the presence of p22-V5, while Ty1*his3-AI-I201T* mobility only decreased 5-fold, confirming the CNC^R^ phenotype (Table 3). Utilizing the V5 tag on p22/p18, co-immunoprecipitations (co-IP) were performed from total cell extracts and analyzed for the level of Gag. We detected co-IP of p22-V5/p18-V5 with wild type Gag (S2 Fig), which confirms previous pull-down analyses with p18 tagged with GST [26]. p22-V5/p18-V5 co-immunoprecipitated 1201T (S2A Fig), K186Q (S2B Fig) or wild type Gag with comparable efficiencies.

**Table 3:** CNC-resistant pGTy1*his3-AI* mobility when co-expressed with p22-V5.

DG3508 (Ty1-less)	pGTy1*his3-AI* mobility x 10^−4^ (SD)	Fold decrease
+ pGTy1*his3-AI*,	160 (19)	1
+empty		
+pGTy1*his3-AI*,	0.2 (0.03)	783
+pGp22-V5		
+ pGTy1*his3-AI-I201T*,	310 (34)	1
+empty		
+ pGTy1*his3-AI-I201T*,	61 (19)	5
+pGp22-V5		

### I201T VLP assembly excludes p22

**Fig 7:**
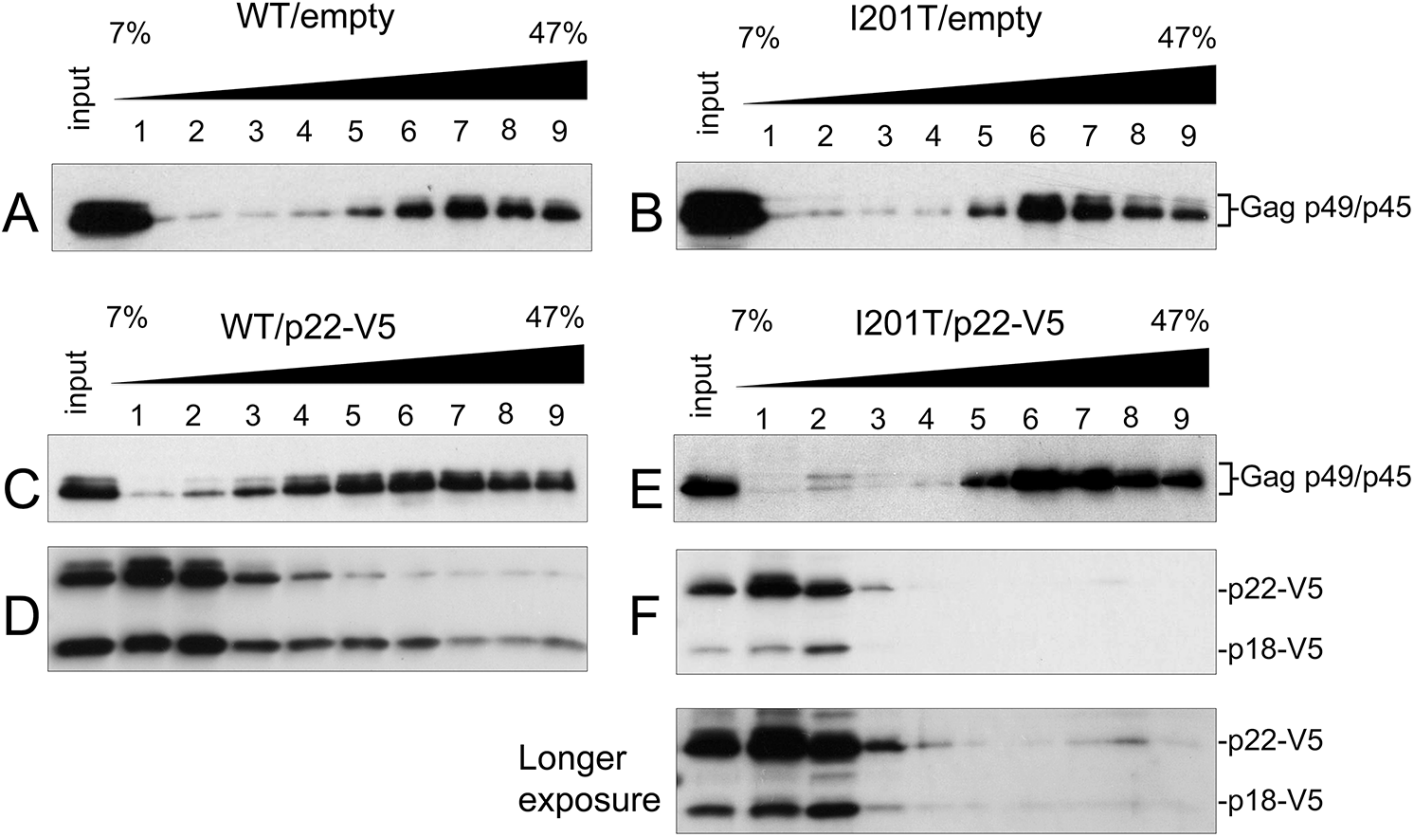
I201T CNC^R^ VLP assembly excludes p22-V5. Protein extracts (input) from *S*. *paradoxus* strains (DG3508) co-expressing WT (pBDG1534) or pGTy1*his3-AI-I201T* (pBJM24) and an empty vector (pRS416; A, B) or p22-V5 (pBJM93; C-F) were centrifuged through a 7–47% continuous sucrose gradient. Equal volumes of each fraction were immunoblotted with p18 (A-C, E) and V5 antibodies (D, F).

To track Gag and p22 and p18 independently during VLP assembly, the fractionation pattern of Gag was examined by sucrose gradient sedimentation as in Fig 4C using total protein extracts from cells expressing wild type pGTy1*his3-AI* or pGTy1*his3-AI-I201T* alone or co-expressed with pGp22-V5 (Fig 7). In the absence of p22-V5, wild type and I201T Gag-p49/p45 assembled into VLPs and migrated to fractions 6–9 (Fig 7A and 7B). In the presence of p22-V5, the fractionation pattern of wild type Gag was more dispersed, as reported previously [26] (Fig 7C). While Gag was present throughout the gradient, it was found at the highest concentration in fractions 4–9. In contrast, p22-V5 when co-expressed with wild type pGTy1*his3-*AI collects as both p22-V5 and p18-V5 and was present in the highest concentration at the top of the gradient (Fig 7D). More p18-V5 co-sedimented with wild type Gag than p22-V5, but p22-V5 and p18-V5 were detected in all fractions. The co-sedimentation of wild type Gag and p18-V5 supports the idea that cleavage by Ty1 PR occurs in complexes migrating to the lower half of the gradient. Surprisingly, p18-V5 was also present at the top of the gradient, which contains most of the soluble proteins in the extract. Considering that the introduction of the internal V5 tag does not alter the requirement of Ty1 PR for cleavage (S3 Fig), p22-V5 may be cleaved by Ty1 PR outside of fully assembled VLPs, perhaps in the Gag complexes present in retrosomes. Alternatively, p22-V5 may be cleaved within VLPs, but not all p22-V5/p18-V5 remains stably associated with the particles. Regardless, our results suggest that a fraction of p22-V5/p18-V5 co-assembles with wild type VLPs. Expression of pGTy1*his3-AI-I201T* and pGp22-V5 resulted in a Gag fractionation pattern similar to that observed in the absence of p22-V5 (Fig 7E). Interestingly, p22-V5/p18-V5 was detected at the top of the gradient but did not co-sediment with I201T VLPs (Fig 7F). These results suggest that the CNC^R^ conferred by I201T results from the exclusion of p22-V5/p18-V5 from VLPs, perhaps during a step in the assembly process. However, the fact some p18-V5 is produced in these cells suggests that the restriction factor does gain access to PR.

## Discussion

To understand the mechanism of inhibition of Ty1 retrotransposition by the self-encoded restriction factor p22, we isolated and characterized Ty1 element CNC^R^ mutants. All but one of the recovered resistance mutations mapped within *GAG* and altered Gag protein sequence. More than half of the mutations mapped outside of p22 coding sequence, including the three strongest CNC^R^ mutations recovered (N183D, K186Q, and I201T). Importantly, most CNC^R^ mutations do not reflect simple gain-of-function since the mutations do not increase Ty1 mobility in the absence of p22 (Fig 2B). These results, along with the observations that the mutant centromere-based pGTy1*his3-AI* plasmids do not produce detectable p22 levels (Fig 1B) or confer CNC [22] due to their low copy number, demonstrate that Gag is the primary molecular target of p22. Although we focused on *GAG* mutations as they represent the vast majority of resistance mutations recovered, one CNC^R^ mutant contains two sequence changes (D518G/V519A) in the Ribonuclease H (RH) domain of RT (D518G/V519A) within *POL* that dramatically affected Ty1 fitness in the absence of p22 (0.4% recovery of wild type mobility). The RH domain of RT is responsible for degrading the RNA template during reverse transcription (reviewed in [66]), and the decrease in Ty1 mobility is probably due to the fact that D518 is a conserved residue predicted to be involved in metal chelation [67]. Mutations resulting in decreased Ty1 RT activity, including active site mutations within the polymerization domain or host mutations that inhibit RT activity by altering cytoplasmic manganese levels can be suppressed by mutations in the RH domain [67, 68]. This suppression has been attributed to allosteric communication between the RT polymerization domain and the RH domain [67]. Full-length cDNA is not detectable in cells undergoing CNC [22], because there is a low level of the initial reverse transcription product minus strand strong-stop DNA [69]. The failure of RT is likely a downstream effect of the alteration in VLP maturation and the absence of mature IN [22, 26]. Although the resistance mutation in RH may bypass the primary defect imposed by p22, it may promote a conformation of RT/RH that allows a low level of activity.

The data presented here greatly extends previous work suggesting that a Gag/p22 interaction is central to the mechanism of CNC [26, 27]. Strains undergoing CNC experience a decrease in Ty1 retrotransposition as Ty1 copy number increases [20, 22, 26]. A decrease in mobility of a genomic Ty1*his3-AI* element was not observed when additional genomic copies carry CNC^R^ mutations, indicating that mutations in Gag, including those that map outside of p22 coding sequence, can relieve CNC in a genomic context. We have not identified resistance mutations with greater than 30% recovery of Ty1 mobility without additionally affecting the fitness of the element (Fig 2). Similarly, combining CNC^R^ mutations resulted in a loss of Ty1 fitness, rather than a combinatorial increase in resistance. These results illustrate the delicate balance between resistance to p22 and overall fitness of Ty1. A similar tradeoff between resistance and fitness exists for mutations in HIV–1 CA that confer resistance to the restriction factor TRIM5α [40]. The inability to achieve complete resistance to TRIM5α is attributed to the genetic fragility of HIV–1 CA, meaning that it is highly sensitive to mutation, and the fact that TRIM5α can bind multiple surfaces on the CA lattice [40, 49]. Similarly, some CA mutations conferring resistance to the Mx2 restriction factor also have a negative impact on HIV infectivity [41]. Ty1 Gag is a multifunctional protein but unlike retroviral Gag is not cleaved into the functionally distinct proteins such as matrix, CA, and nucleocapsid (NC). Even so, Ty1 Gag is responsible for executing the same functions as retroviral CA and NC. Thus our inability to obtain fully resistant Ty1 elements strongly suggests some of the same rules concerning genetic fragility apply to Ty1 Gag, namely that its function is very sensitive to mutation. Secondly, p22 may bind multiple surfaces of the VLP during different stages of assembly, making it difficult to attain full resistance by mutating Gag at only one or two residues. A third consideration is that the surfaces or protein domains that interact with p22 may be the same or overlap with domains important for Gag function. In support of this idea, we recovered Gag mutations, P173L and K250E, that confer CNC^R^, yet negatively impacted Ty1 fitness in the absence of p22 (Fig 2). K250 is located within predicted helix 4, an amphipathic helix important for VLP maturation (Fig 3A) and perturbation at this site may prevent p22-mediated effects, perhaps by altering VLP assembly and maturation dynamics. However, this alteration in VLP function was not efficient in the absence of p22. Like other infectious agents, the presence of Ty elements in *Saccharomyces* has resulted in positive selection for certain host genes, suggesting there is an ongoing “genetic conflict” or evolutionary arms race between Ty elements and their host [70]. In our screen, we recovered mutations that mapped within Gag but not p22 coding sequence and found it difficult to recover mutations that fully restored Ty1 retrotransposition in the presence of p22. Gag and p22 share coding sequence in the natural setting, and this is likely to influence a Ty1-p22 arms race for the adaptation of Ty1 to inhibition via p22.

Our bioinformatic analysis of Ty1 Gag revealed 9 predicted helical stretches and two Pfam domains: TYA in the N-terminal half and UBN2 in the C-terminal half of the protein (Fig 3A). Four CNC^R^ mutations mapped within UBN2; although no CNC^R^ mutations were isolated in TYA. UBN2 is a Gag sequence motif that is found in Ty1/*copia* retrotransposons across plants and fungi. Because UBN2 can be recognized by profile-based methods across a wide range of organisms, this domain is likely involved in a conserved function of Gag. Because known mutations that affect VLP assembly fall within this domain [55], it is reasonable to hypothesize that the UBN2 domain is involved in VLP assembly. UBN2 also overlaps with, but does not fully encompass, the NAC region of Gag (Fig 3A) [28]. Recent work demonstrates that p18 interferes with Gag NAC function [27]. It would be interesting to investigate whether CNC^R^ mutations modulate NAC activity of Gag, although we showed that I201T VLPs do not exhibit enhanced levels of Ty1 RNA packaging (Fig 6E). Additionally, only V336I and Q350R S395L mapped within the NAC region (S2 Table, Fig 3A), suggesting that CNC^R^ does not primarily alter Gag NAC functions. The remaining CNC^R^ mutations mapped to a central region of Gag, which we named the CNC^R^ domain. Predicted helix 1 within the CNC^R^ domain was frequently mutated in our screen and overlaps with conserved domain A present in all Ty1/*copia* elements that surrounds an invariant tryptophan (Gag W184 for Ty1) [53]. Ty2 Gag differs from Ty1 Gag at many positions and some CNC^R^ mutations are present within Ty2 sequences, raising the possibility that Ty2 is naturally resistant to Ty1 CNC. Ty1 and Ty2 are closely related retrotransposons based on their near identical LTR sequences, with a single nucleotide deletion defining Ty2 LTR sequences [2, 71]. Phylogenetic analyses suggest that *S*. *cerevisiae* recently acquired Ty2 elements from *S*. *mikatae* by horizontal transfer [3, 72]. We showed that Ty2-917 is neither under CNC by Ty2-917 nor inhibited by *GAL1*-promoted expression of p22 from Ty1. CNC^R^ residues are also altered in a Tsk1 element from *L*. *kluyveri* [57] and a Ty1-like element present in S. *kudriavzevii* and hybrids thereof [3, 56]. Further mutational analyses of specific CNC^R^ residues within these elements will be required to address the role of naturally occurring CNC^R^ residues in Ty1 and Ty2. In addition, the overlap between Gag and p22 coding sequence may create specificity even within Ty1 elements. It will be interesting to determine if Ty1 elements in natural *Saccharomyces* isolates confer CNC on Ty1-H3 or whether they have evolved specificity for elements in their native genomes.

Though the structural role of CNC^R^ residues within Ty1 Gag’s helical domains remains to be determined, the mutational analyses presented here define the importance of key hydrophobic residues within these predicted regions. Mutation of the conserved W184 residue within helix 1 to alanine resulted in complete loss of transposition and formation of mature Pol proteins, as well as abnormal VLP assembly (Fig 4). Other Gag helix mutations we tested were previously analyzed in the context of a truncated Gag protein containing amino acids 1–381, which is deleted for 21 residues at the C-terminus of p45 and lacks a complete NAC domain [28, 55]. The truncated Gag is still able to form particles and thus was used to address the role of certain residues in particle assembly. In the context of 1–381 particles, L252R was reported to completely disrupt particle assembly, while LF339-340RD has no effect. IM248/249NR and I343K both alter the migration of 1–381 particles through a sucrose gradient and assemble into “giant” particles when visualized by negative staining and TEM. These mutations had not been characterized in the context of a full length Ty1 element nor analyzed for effects on Ty1 mobility. Therefore, our work showed that altering these hydrophobic residues within helices severely hinders Ty1 mobility (Fig 4). Our results regarding particle assembly with these Gag substitutions differ from previous work, as L252R was capable of forming higher order complexes and I343K was not (Fig 4C). These conflicting results could be due to the differences in multimerization and VLP assembly using full length Gag compared to truncated 1–381 Gag used by others [55]. Importantly, since most CNC^R^ mutations mapped to helices required for normal Ty1 transposition, protein processing and VLP assembly, our results indicate that inhibition by p22 disturbs a central function of Gag.

Mutations exhibiting the highest level of CNC^R^ (N183D, K186Q, I201T, and A273V) were associated with reduced levels of p18 (Fig 5), which is cleaved from p22 near its C-terminus by Ty1-PR, and there was less co-assembly between p22 and Gag I201T compared to wild type Gag (Fig 7). These results suggest that the reduction in p18 levels in the presence of the CNC^R^ VLPs may result from diminished access to PR. More p22/p18 associated with purified wild type VLPs when compared with I201T VLPs (Fig 6D). When total cellular protein from cells co-expressing p22-V5 and wild type or Ty1*his3-AI-I201T* was analyzed by sucrose gradient sedimentation (Fig 7), the majority of p22-V5 and p18-V5 remained in fractions containing soluble protein rather than in fractions containing VLPs or higher order assembly intermediates. Because Ty1 PR-mediated processing is thought to occur only within assembled VLPs and cleavage of p22 and p22-V5 was Ty1 PR-specific (Fig 1D and S3 Fig), p22/p18 may be capable of moving in and out of the VLP. Perhaps this occurs by diffusion of p22/p18 through VLP pores, which are permeable to ribonuclease A (15.7 kD) but not to benzonase (30 kD) treatment *in vitro* [73–75]. Consequently, p22/p18 may still be within the acceptable size limit to enter and exit VLP pores. Alternatively, once p22/p18 co-assembles with Ty1 proteins in VLPs and maturation is initiated, p22/p18-containing VLPs may be subject to dissociation and degradation. Although we did observe a modest shift in Ty1 Gag fractionation towards the top of the gradient in the presence of p22/p18, Gag was not concentrated in the first two fractions with p22/p18. Lastly, our results raise the possibility that Ty1 PR may function outside of stably assembled VLPs, perhaps in assembly intermediates present in retrosomes, which are cytoplasmic foci containing Ty1 mRNA and proteins [7, 9, 76]. In support of this idea, few if any VLPs are detected in cells containing retrosomes resulting from endogenous Ty1 expression, VLP assembly increases dramatically when Ty1 is overexpressed from a strong promoter, and assembly occurs within retrosomes [7, 25, 77]. Recent work also shows that steady state Gag expressed from endogenous Ty1 elements does not co-migrate with unprocessed Gag-p49 [76], suggesting that Gag cleavage can occur in the absence of detectable VLPs. Finally, several earlier studies demonstrate the presence of mature p45 resulting from endogenous Ty1 expression [8, 24, 78–80]. Together, our results suggest that p22 cleavage may occur in the same spatiotemporal environment as pre-VLP Gag cleavage.

We observed varying degrees of p22 cleavage and/or p18 stability in the presence of altered Ty1 Gag proteins. While p22/p18 levels were comparable to WT in Gag L252R, p18 was not detected in Gag LF339-340RD and Gag I343K. CNC^R^ mutations (N183D, K186Q, I201T, and A273V) and the helix-altering Gag W184A and IM248-249NR were associated with decreased levels of p18, but we cannot distinguish if these changes represent a decrease in p22 cleavage or a reduction in p18 stability. It is interesting to consider that some loss-of-function changes in Gag (W184A and IM248-249NR) and the gain-of-function CNC^R^ mutations both result in decreased p18 levels. Perhaps p22 cleavage is a read-out for both Ty1 PR activity, which can be affected by several different situations, such as Gag:Gag-Pol ratio and particle assembly [47, 81–83], or access of the p22 substrate to Ty1 PR. Whereas the helix mutations alter VLP assembly, the resistance mutations likely affect access to PR, since p22 is excluded from CNC^R^ VLPs. Thus, reduced p22 cleavage can occur in both loss-of-function and gain-of-function contexts.

Although the CNC^R^ mutations in Gag might affect Gag/p22 binding, co-IP experiments performed using standard washing conditions did not support a simple interaction between p22 and Gag involving CNC^R^ residues (S2 Fig). In addition, sucrose gradient fractionation indicated that most p22-V5/p18-V5 was present in the fractions containing soluble proteins and did not co-sediment with VLPs. Perhaps p22 is capable of binding several forms of Gag, whether monomeric, small assembly intermediates, or intact VLPs, and perhaps these interactions inhibit VLP assembly or maturation with different potencies. If the crucial binding substrate of p22 is multimeric and represents a minority of Gag molecules present in the cell, co-IP analysis may not show differences in binding. Interestingly, retroviral CA-binding restriction factors TRIM5α and the Gag-derived Fv1 bind to their CA target after polymerization of the lattice [84, 85]. We are considering that the interaction between p22 with polymerized/assembled Gag alone may be the defining and initial insult to Ty1 replication.

Retroviral studies involving sensitivity and escape from host restriction factors show similarities to the Ty1-p22 system. Mx2 restriction of HIV–1 is thought to involve inhibition of viral uncoating and/or nuclear entry and requires Mx2-CA binding [41, 86]. However, known Mx2 escape mutations in the CA gene do not significantly alter binding between Mx2 and CA [41], which demonstrates that viral escape mutations can promote replication in ways distinct from the disruption of restriction factor-target binding. In the case of the resistant provirus enJSRV26, increasing the levels of enJSRV26 Gag expression relative to the restriction factor enJS56A1 Gag protein is enough to allow JSRV replication in sheep [43]. Increased expression of enJSRV–26 Gag is achieved by mutation of the signal peptide in the envelope glycoprotein, which modulates proviral gene expression. Similarly, increasing the level of Ty1 expression can overcome CNC [48]. We favor the hypothesis that understanding how p22 is excluded from CNC^R^ VLPs is central to understanding CNC. Since the steady state level of Gag was unaffected in CNC^R^ mutants (Fig 5A), perhaps the ratio of Gag:p22 is specifically higher within retrosomes comprised of CNC^R^ Gag.

In summary, we have shown that mutations in Gag confer resistance to the p22 restriction factor produced by Ty1 during CNC. These mutations are beneficial only in the presence of p22 and do not globally increase Ty1 mobility. CNC^R^ mutations allow for VLP maturation, which may be the step in Ty1 replication most sensitive to CNC, by excluding p22 from assembling particles. Identification of the Gag multimerization states that bind p22 and host factors that modulate Gag assembly, in combination with studies examining VLP assembly dynamics and structure, especially regarding the newly identified Gag domains, will deepen our understanding of retroelement control.

## Materials and Methods

### Genetic techniques, media and strain construction

Strains are listed in S1 Table. Strains repopulated with Ty1 elements were obtained following pGTy1 induction as described previously [20]. Ty1 insertions following repopulation experiments were estimated by Southern blotting as in [48]. Standard yeast genetic and microbiological procedures were used in this work [87].

### Plasmids

Refer to S3 Table for plasmid descriptions and sources. Directed mutagenesis was carried out by overlap PCR using the following primer sequences: W184Ab; 5’-ATGTTTTAACAGCATTTGGAAAGTCATTAGGTGAGGTTAAC; W184Ac, 5’-GACTTTCCAAATGCTGTTAAAACATACATCAAATTTTTAC; L252Rb, 5’-ATACTTTTGGATCTAATTTTCATGATATCCGTATAATCAACG; L252Rc, 5’-TCATGAAAATTAGATCCAAAAGTATTGAAAAAATGCAATCTG; IM248/9NRb, 5’-AAAGAATTTTCCTGTTATCCGTATAATCAACGGATAGGAT; IM248/9NRc, 5’-TATACGGATAACAGGAAAATTCTTTCCAAAAGTATTGAAA; LF339/40RDb, 5’-GGATATCTAAGTCCCGTTCAGCGACTGTCATATTTAGATG; LF339/40RDc, 5’-GTCGCTGAACGGGACTTAGATATCCATGCTATTTATGAAG; I343Kb, 5’-AAATAGCATGCTTATCTAAGAACAGTTCAGCGACTGTCAT; I343Kc, 5’-CTGTTCTTAGATAAGCATGCTATTTATGAAGAACAACAGG. For pBJM24, the plasmid markers were switched from *URA3* to *TRP1*, as described previously [26]. Galactose-inducible centromere (*CEN*) vectors expressing p22-V5 were created by PCR amplification of Ty1-H3 p22 coding sequence 1038–1613 with the internal V5 tag and flanking *GAL1P* and *CYC1* TT sequences using pBDG1568 as a template [26] and primers: cla1_galp, 5’-CATGTTTCATCGATACGGATTAGAAGCCGCCGAGC; cyc1ttrevSacII, 5’-CATGTTTCCCGCGGGAGTCAGTGAGCGAGGAAGC. The insert was cloned into an empty *URA3*/*CEN* vector (pRS416) using ClaI and SacII sites. The V5 tag is located between nucleotides 1442 and 1443[26]. pTy2-917*his3-AI* (pBDG631) was constructed by digestion of pGTy917 with BglII and pBJC42 (*his3-AI*, pBDG619) with ClaI, fill-in synthesis of the linearized vector and *his3-AI* fragments using DNA polymerase I followed by blunt end-ligation. pGPOLΔ*d1* was constructed by BglII digestion and reclosure of pGTy1*his3-AId1* (kindly provided by Jef Boeke [54, 64] and Joan Curcio [88]), which deletes the majority of *POL*. pYES2-p45 was constructed by PCR using primers specific for the coding sequence of p45 and the amplification product was cloned into the multi-copy *GAL1*-promoted expression vector pYES2 [27]. Recombinant plasmids were verified by restriction enzyme analysis or DNA sequencing.

### CNC-resistance screen

Plasmid mutagenesis was performed by transforming 50 ng of pBDG606 (S3 Table) into XL–1 Red (Agilent Technologies) cells and sub-culturing transformants for 3–4 days at 37°C. Gap repair was performed with pBDG606 using mutagenized *GAG* template and AatII (upstream of *GAL1*P) and BstEII (within PR) sites, while *POL* mutagenesis was performed using BstEII and XbaI (within *his3-AI*) sites. Primers flanking these restriction sites (*GAG*: USAatII, 5’-ATAATACCGCGCCACATAGC; RP1, 5’-CATTGATAGTCAATAGCACTAGACC; *POL*: USBsteIIf, 5’-GCACGACCTTCATCTTAGGC; 3pLTRrev, 5’-ATCAATCCTTGCGTTTCAGC) were used in a standard *Taq* (ThermoFisher Scientific, Waltham, MA) PCR reaction with Ty1-H3 as a template to mutagenize the area of interest at a low frequency. XL–1 Red treated pBDG606 or DNA fragments for gap repair were transformed into YEM515 (see S1 Table) and plated onto SC-Ura. Transformants, were replica plated on SC-Ura + 2% galactose, incubated at 22°C for 1–2 days, and then replica plated on SC-Ura-His and incubated at 30°C for 2 days. Candidate plasmids were extracted, propagated in *E*. *coli*, transformed into YEM514 and YEM515 and retested for pTy1*his3-AI* mobility. Candidates with at least a 10-fold increase in retromobility in YEM515 were carried forward. After sequencing the CNC region of XL–1 Red treated plasmids, the *GAL1* and *GAG* segments were subcloned into wild type plasmid using AatII and Eco91I sites to eliminate other mutations present outside of the region of interest. In all cases, subcloned *GAG* mutations conferred a similar level of CNC^R^ as the primary isolates. For the gap repair screen, the entire region amplified by low fidelity PCR was sequenced.

### Ty1*his3-AI* mobility

The mobility frequency of Ty1*his3-AI* was determined as described previously [21, 48] with the following modifications. For strains transformed with only pGTy1*his3-AI*, single colonies were grown at 30°C overnight in 1 ml of SC-Ura + 2% raffinose and then diluted 1:25 in quadruplicate 1 ml SC-Ura + 2% galactose cultures. Galactose cultures were grown at 22°C for 2 days, and cells were then washed, diluted and spread onto SC-Ura and SC-Ura-His plates. For strains transformed with both pGTy1*his3-AI* and p22-containing plasmids, similar methods were used for the assay except liquid and solid media also lacked tryptophan. For qualitative mobility assays with pGTy1*his3-AI*, cells were patched onto SC-Ura and grown at 30°C for 2 days. Cells were replica plated onto SC-Ura +2% galactose and incubated at 22°C for 2 days, followed by replica plating onto SC-Ura-His and incubation at 30°C until His^+^ papillae appeared. For transposition assays involving chromosomal Ty1*his3-AI*, a single colony was dissolved in 10 ml water. One microliter of diluted cells was added to quadruplicate 1 ml SC-Ura or YEPD cultures and grown 2–3 days until saturation. The cells were washed, diluted and spread onto SC-Ura or YEPD and SC-Ura-His or SC-His plates, and incubated at 30°C until colonies formed.

### Protein isolation and immunoblotting

For strains carrying pGTy1*his3-AI*, 1 ml of SC-Ura + 2% raffinose was inoculated with a single colony and grown overnight at 30°C, then diluted 1:10 into SC-Ura + 2% galactose and grown at 22°C for 24 h. For growth in glucose, a dilution of 1:100 was used. To detect p22/p18, 5 ml of culture was processed by trichloroacetic acid (TCA) extraction as described previously [26]. To detect all other Ty1 proteins, protein from 10 ml of culture was extracted as previously described [89] and 30 μg of protein was used for immunoblotting. Samples were separated on 10% (for RT and IN detection or to separate Gag-p49 and p45) or 15% (Gag-p49/p45 and p22/p18 detection) SDS-PAGE gels and immunoblotted as described previously [26]. Antibody dilutions were as follows: anti-p18 1:5000 [26], anti-VLP 1:10,000, anti-RT 1:5,000, anti-IN 1:2500, anti-Hts1 1:40,000, anti-TY (BB2, UAB Epitope Recognition and Immunoreagent Core, Birmingham AL) 1:50,000, anti-V5 1:20,000 (Life Technologies, Carlsbad, CA).

### Analysis of Ty1 Gag sequence

Ty1-H3 sequence (GenBank M18706.1) was submitted to the following online servers for secondary structure prediction: ITASSER [50–52] PredictProtein [90], Sable [91], PSIPRED [92], and SAMTO8[93]. When comparing the secondary structure predictions, the results were consistent, with the same helices predicted by all five servers. The boundaries of the helices varied slightly, but not by more than three residues. The I-TASSER results were chosen for display in Fig 3. Protein domains in Ty1 sequence were predicted using profile hidden Markov models [94] by scanning Ty1 Gag sequence against the Pfam database. Ty1 related sequences in UniProt were identified using HMMER [94] and aligned using CLUSTALW [95]. Full alignment can be found in the supplemental data (S1 Fig). Protein alignments were visualized using Jalview (http://www.jalview.org/) [96]. ClustalX coloration was used with a conservation color increment of 35. The raw alignment file is provided as S1 File.

### VLP isolation

VLPs were isolated as described previously [26], except the cells were induced in SC-Ura + 2% galactose for 24 h at 20°C. Two micrograms of final VLPs were immunoblotted to detect Gag, RT, and IN. A 1:2 dilution series was loaded to detect p18.

### RNA isolation and northern blotting

Equivalent total cellular RNA and VLP RNA, as estimated by OD_600_ or total Gag protein respectively, was extracted using the MasterPure yeast RNA purification kit (Epicentre Biotechnologies, Madison, WI) and analyzed via Northern blotting as previously described [26].

### V5 immunoprecipitation

Antibodies were crosslinked to resin using a Pierce Crosslink IP Kit (ThermoFisher Scientific) and following the manufacturer’s instructions. For immunoprecipitations, a 25 ml culture was induced in SC-Ura-Trp + 2% galactose at 20°C for 24 h or until OD_600_ = 1.0. IP Lysis buffer was supplemented with 1 μg/ml aprotonin, pepstatin and leupeptin and 1 mM PMSF. Cells were broken in IP Lysis buffer plus protease inhibitors by vortexing with glass beads. Equal amounts of protein were applied to Protein A/G agarose crosslinked with 2 μg of V5 Antibody (Life Technologies) and allowed to bind for 2 h at 4°C. Beads were washed with IP Lysis buffer and eluted with 20 μl of elution buffer. 1/100 of the input and 1/2 of the pull-down material were loaded per lane. Beads not crosslinked to V5 antibody served as a negative control.

### Sucrose gradient sedimentation

A 100 ml culture was induced in SC-Ura or SC-Ura-Trp + 2% galactose at 20°C for 24 h or until the culture reached an OD_600_ of 1. Cells were broken in 15 mM KCl, 10 mM HEPES-KOH, pH 7, 5 mM EDTA containing RNase inhibitor (100 U per ml), and protease inhibitors (16 μg/ml aprotinin, leupeptin, pepstatin A and 2 mM PMSF) in the presence of glass beads. Cell debris was removed by centrifuging the broken cells at 10,000 x g for 10 min at 4°C. Five milligrams total protein in 300–500 μl of buffer was applied to a 7–47% continuous sucrose gradient and centrifuged using an SW41 rotor at 25,000 rpm (~100,000 x g) for 3 h at 4°C. After centrifugation, 9 x 1.2 ml fractions were collected and 30 μg of the input and 15 μl of each fraction was immunoblotted to detect Ty1 proteins.

## Supporting Information

S1 Table Yeast strains. (PDF)

S2 Table CNC-resistant mutations. (PDF)

S3 Table Plasmids. (PDF)

S4 Table CNC^R^ pGTy1*his3-AI* mobility. Ty1-less *S*. *paradoxus* strains repopulated with 1 (YEM514) or 38 (YEM515) copies of Ty1-H3 and containing wild type or CNC^R^ pGTy1*his3-AI* were analyzed for Ty1*his3-AI* mobility as described in the Materials and Methods. *Ty1his3-AI* mobility is calculated by dividing the number of colonies that form on SC-Ura-His by number of colonies on SC-Ura, and the standard deviation (SD) from 4 replicates is shown. Frequencies from individual experiments are shown, but the entire analysis was repeated at least three times with comparable results.
(PDF)

S5 Table Ty2-917*his3-AI* mobility. A Ty1-less *S*. *paradoxus* strain with Ty2-917*his3-AI* (DG3664) was transformed with the following multi-copy plasmids (2μ): (A) empty (pRS416), pGTy2-917 (pGTy2-917) or pTy2-917 (pJC384) (B) empty (pYES2) or p22 (pBDG1565). Cells were grown at 22°C for two days in SC media containing glucose (A) or galactose (B). Numbers represent Ty2*his3-AI* mobility events per cell and standard deviations are provided in parentheses. Mobility assays were repeated at least three times and representative results are shown.
(PDF)

S6 Table PR/p4 is not required for CNC. A Ty1-less *S*. *paradoxus* with a single Ty1*his3-AI* (DG2196) was transformed with empty vector pGAL (pBDG101), wild type pGPOLΔ (pBDG1130), pGPOLΔ*d1* (pBDG1586), or pYES2-p45 (pBDG1375). pGPOLΔ plasmids are deleted for most of *POL*, produce p22 and confer CNC [26]. The *d1* deletion in PR/p4 has been characterized extensively and alters a PR-specific activity involved in reverse transcription and not proteolysis [54, 64]. Note that we are testing for a p22-specific role of PR/p4 sequence, as wild type PR is not produced by pGPOLΔ plasmids due to truncation of *POL*. The *GAG* ORF of pYES2-p45 ends at the mature C-terminus of Gag, and thus contains a complete deletion of p4 sequence. Cells were grown in glucose and numbers represent Ty1*his3-AI* mobility events per cell. Standard deviations are provided in parentheses.
(PDF)

S1 Fig Full alignment of yeast Ty1-like Gag sequences. The alignment was generated with ClustalW and visualized with Jalview using the ClustalX color scheme. Uniprot annotations are included to the left and the following domains, which are described in the main text, are labeled: TYA (PF01021), CNC^R^, and UBN2 (PF14223). Ty1-H3 (P08405) and Ty2-917 were included, however, these elements were isolated as spontaneous retrotransposition events and are unique from known genomic elements [58, 59].
(TIF)

S2 Fig WT and I201T Gag co-immunoprecipitate with p22-V5. Protein extracts (input) from Ty1-less *S*. *paradoxus* strains (DG3508) co-expressing WT (pBDG1534) or pGTy1*his3-AI-I201T* (A) or pGTy1*his3-AI-K186Q* (B) and p22-V5 (pBJM93) were incubated with Protein A/G Agarose beads crosslinked to V5 antibody for 2 hours at 4°C. After washing, bound proteins were eluted and immunoblotted with p18 and V5 antibodies. Beads not crosslinked to V5 antibody and Hts1 were used as controls.
(TIF)

S3 Fig Cleavage of p22-V5 to p18-V5 is Ty1 PR-specific. *S*. *paradoxus* (DG3508) expressing p22-V5 (pBJM93) was transformed with empty vector (pRS414), wild type (pBDG1534), or PR-defective (PR^-^) pGTy1*his3-AI* (pBDG1606). TCA-precipitated extracts were immunoblotted with p18, V5, and Hts1 antibodies. PR was inactivated via a SacI linker insertion at the BglII site in Ty1-H3 [47].
(TIF)

S1 File Alignment file of yeast Ty1-like Gag sequences. The alignment displayed in S1 Fig provided in ClustalW format.
(CLUSTALW)
