# Supplementary material for: The Ty1 Retrotransposon Restriction Factor p22 Targets Gag
Source: PLoS Genet. 2015 Oct 9;11(10):e1005571. doi: 10.1371/journal.pgen.1005571 (PMC4599808; doi:10.1371/journal.pgen.1005571)
Supplement: S1 Table — (PDF) [file pgen.1005571.s001.pdf]

S1 Table. Yeast Strains.

Strain	Relevant Genotype	Genomic Ty1s	Source
YEM514	*MATα*, *his3-Δ200hisG*, *ura3*, *spt3-ΔKanMX4*	Ty1-less, +1 Ty1-4253*his3-AI*	(1)
YEM570	YEM514	+7 Ty1	E. Matsuda
YEM568	YEM514	+14 Ty1	E. Matsuda
YEM572	YEM514	+21 Ty1	E. Matsuda
YEM515	YEM514	+37 Ty1	(1)
DG3582	*MATα*, *his3-Δ200hisG*, *ura3*, *trp1*	Ty1-less	(2)
DG3710	DG3582	+2 Ty1*his3-AI*	This study
DG3716	DG3582	+2 Ty1*his3-AI-N183D*	This study
DG3713	DG3582	+1 Ty1*his3-AI*	This study
DG3725	DG3582	+1 Ty1*his3-AI-K186Q*	This study
DG3719	DG3582	+1 Ty1*his3-AI-A273V*	This study
DG3722	DG3582	+1 Ty1*his3-AI-I201T*	This study
DG2533	*MATα*, *his3-Δ200hisG*, *ura3*	Ty1-less, +1 Ty1-4253*his3-AI*	(3)
YEM13	DG2533	+14 Ty1	E. Matsuda
YEM14	DG2533	+21 Ty1	E. Matsuda
JM487	DG2533	+18 *Ty1-K186Q*	This study
JM506	DG2533	+20 *Ty1-K186Q*	This study
JM484	DG2533	+14 *Ty1-I201T*	This study
JM503	DG2533	+19 *Ty1-I201T*	This study
DG3508	DG3582, *spt3-ΔKanMX4*	Ty1-less	This study
DG3664	DG3582	Ty2-917*his3-AI*	This study
DG2196	MATa, *his3-Δ200hisG,* ura3, trp1	Ty1-less, +1 Ty1*his3-AI(96)*	(4)

Supplemental References for Strains

1. Matsuda E, Garfinkel DJ. Posttranslational interference of Ty1 retrotransposition by antisense RNAs. Proc Natl Acad Sci U S A. 2009;106(37):15657-62.
2. Saha A, Mitchell JA, Nishida Y, Hildreth JE, Ariberre JA, Gilbert WV, et al. A trans-Dominant Form of Gag Restricts Ty1 Retrotransposition and Mediates Copy Number Control. J Virol. 2015;89(7):3922-38.
3. Garfinkel D. Genome evolution mediated by Ty elements in Saccharomyces. Cytogenet Genome Res. 2005;110(1-4):63-9.
4. Garfinkel DJ, Nyswaner K, Wang J, Cho J-Y. Post-transcriptional cosuppression of Ty1 retrotransposition. Genetics. 2003;165(1):83-99.
