# Supplementary material for: The Ty1 Retrotransposon Restriction Factor p22 Targets Gag
Source: PLoS Genet. 2015 Oct 9;11(10):e1005571. doi: 10.1371/journal.pgen.1005571 (PMC4599808; doi:10.1371/journal.pgen.1005571)
Supplement: S2 Table — (PDF) [file pgen.1005571.s002.pdf]

S2 Table. CNC-resistant mutations.

Amino acid change	Mutagenesis
Gag T55A Q351L	PCR-based (*GAG*)
Gag P173L	XL-1 Red
Gag M174I R362G	PCR-based (*GAG*)
Gag D180N	XL-1 Red
Gag N183D	XL-1 Red
Gag K186Q	XL-1 Red
Gag I189V S411G/ PR S10G	PCR-based (*GAG*)
Gag I201T	XL-1 Red
Gag T218A	XL-1 Red
Gag T218I	XL-1 Red
Gag K250E	PCR-based (*GAG*)
Gag A273V	XL-1 Red
Gag E287T	XL-1 Red and PCR-
Gag V336I	PCR-based (*GAG*)
Gag Q350R S395L	PCR-based (*GAG*)
RT D518G/V519A	PCR-based (*POL)*
