# Supplementary material for: The Ty1 Retrotransposon Restriction Factor p22 Targets Gag
Source: PLoS Genet. 2015 Oct 9;11(10):e1005571. doi: 10.1371/journal.pgen.1005571 (PMC4599808; doi:10.1371/journal.pgen.1005571)
Supplement: S3 Table — (PDF) [file pgen.1005571.s003.pdf]

S3 Table. Plasmids.

Plasmid	Description	Markers	Source
pBDG606	pGTy1*his3-AI*	*URA3*/*CEN*	(1)
pRS416	Empty	*URA3*/*CEN*	(2)
pGTy1-H3Cla	pGTy1	*URA3*/*2µ*	(3)
pJEF1266	pGTy1pr-1702SacI	*URA3*/*2µ*	(4)
pRS426	Empty	*URA3*/*2µ*	(5)
pTy1-ATGfs	Ty1-ATGfs	*URA3*/*2µ*	(6)
pBJM42	pBDG606-*W814A*	*URA3*/*CEN*	This study
pBJM60	pBDG606-*IM248-249NR*	*URA3*/*CEN*	This study
pBJM43	pBDG606-*L252R*	*URA3*/*CEN*	This study
pBJM61	pBDG606-*LF339-340RD*	*URA3*/*CEN*	This study
pBJM62	pBDG606-*I343K*	*URA3*/*CEN*	This study
pBDG1534	pGTy1*his3-AI*	*TRP1*/*CEN*	(7)
pBJM24	pBDG1534-*I201T*	*TRP1*/*CEN*	This study
pBJM93	pGp22-V5	*URA3*/*CEN*	This study
pRS414	Empty	*TRP1*/*CEN*	(2)

pBDG1606	pGTy1PR- *his3-AI*	*TRP1/CEN*	This study
pGTy917	pGTy2-917	*URA3/2μ*	(8)
pJC384	Ty2-917	*URA3/2μ*	(6)
pBDG1565	pGAL-Yes2:1038-1616	*URA3/2μ*	(7)
pBDG101	pGAL	*URA3/2μ*	(9)
pBDG1130	pGPOLΔ	*URA3/2μ*	(7)
pBDG1375	pYES2-p45	*URA3/2μ*	(10)
pBDG1586	pGPOLΔ*d1* (11)	*URA3/2μ*	This study

Supplemental References for Plasmids

1. Dakshinamurthy A, Nyswaner KM, Farabaugh PJ, Garfinkel DJ. BUD22 affects Ty1 retrotransposition and ribosome biogenesis in Saccharomyces cerevisiae. Genetics. 2010;185(4):1193-205.
2. Sikorski R, Hieter P. A system of shuttle vectors and yeast host strains designed for efficient manipulation of DNA in Saccharomyces cerevisiae. Genetics. 1989;122:19-27.
3. Garfinkel D, Mastrangelo M, Sanders N, Shafer B, Strathern J. Transposon tagging using Ty elements in yeast. Genetics. 1988;120(1):95-108.
4. Youngren SD, Boeke JD, Sanders NJ, Garfinkel DJ. Functional organization of the retrotransposon Ty from Saccharomyces cerevisiae: Ty protease is required for transposition. Molecular and cellular biology. 1988;8(4):1421-31.
5. Christianson TW, Sikorski RS, Dante M, Shero JH, Hieter P. Multifunctional yeast high-copy-number shuttle vectors. Gene. 1992;110(1):119-22.
6. Garfinkel DJ, Nyswaner K, Wang J, Cho J-Y. Post-transcriptional cosuppression of Ty1 retrotransposition. Genetics. 2003;165(1):83-99.
7. Saha A, Mitchell JA, Nishida Y, Hildreth JE, Ariberre JA, Gilbert WV, et al. A trans-Dominant Form of Gag Restricts Ty1 Retrotransposition and Mediates Copy Number Control. J Virol. 2015;89(7):3922-38.
8. Garfinkel DJ, Mastrangelo MF, Sanders NJ, Shafer BK, Strathern JN. Transposon tagging using Ty elements in yeast. Genetics. 1988;120(1):95-108.
9. Curcio MJ, Sanders NJ, Garfinkel DJ. Transpositional competence and transcription of endogenous Ty elements in Saccharomyces cerevisiae: implications for regulation of transposition. Molecular and cellular biology. 1988;8(9):3571-81.

10. Nishida Y, Pachulska-Wieczorek, K., Błaszczyk, L., Saha, A., Gumna, J., Garfinkel, D.J., Purzycka, K.J. Ty1 retrovirus-like element Gag contains overlapping restriction factor and nucleic acid chaperone functions. Nucleic Acids Res. 2015:In press.
11. Merkulov GV, Swiderek KM, Brachmann CB, Boeke JD. A critical proteolytic cleavage site near the C terminus of the yeast retrotransposon Ty1 Gag protein. J Virol. 1996;70(8):5548-56.
