# Supplementary material for: The Ty1 Retrotransposon Restriction Factor p22 Targets Gag
Source: PLoS Genet. 2015 Oct 9;11(10):e1005571. doi: 10.1371/journal.pgen.1005571 (PMC4599808; doi:10.1371/journal.pgen.1005571)
Supplement: S4 Table — Ty1-less S. paradoxus strains repopulated with 1 (YEM514) or 38 (YEM515) copies of Ty1-H3 and containing wild type or CNCR pGTy1his3-AI were analyzed for Ty1his3-AI mobility as described in the Materials and Methods. Ty1his3-AI mobility is calculated by dividing the number of colonies that form on SC-Ura-His by number of colonies on SC-Ura, and the standard deviation (SD) from 4 replicates is shown. Frequencies from individual experiments are shown, but the entire analysis was repeated at least three times with comparable results. (PDF) [file pgen.1005571.s004.pdf]

S4 Table. CNC[R] pGTy1*his3-AI* mobility.

**Experiment**	**Ty1**	**pGTy1*his3AI***	**pGTy1his3-AI mobility X 10^{-5} (SD)**
**A**	1	WT	3500 (610)
	38	WT	1.8 (0.5)
	38	Gag N183D	420 (70)
	38	Gag A273V	440 (96)
	38	Gag I201T	620 (110)
	38	Gag K186Q	760 (120)
**B**	1	WT	2100 (300)
	38	WT	3.1 (0.4)
	1	Gag N183D	1900 (250)
	1	Gag A273V	2200 (340)
	1	Gag I201T	2100 (250)
	1	Gag K186Q	2500 (530)
**C**	1	WT	990 (320)
	38	WT	1.3 (0.3)
	38	Gag P173L	85 (12)

	38	Gag D180N	45 (8.6)
	38	Gag T218A	53 (12)
	38	Gag T218I	43 (12)
	38	Gag E287T	43 (16)
**D**	1	WT	2300 (270)
	38	WT	2 (0.3)
	1	Gag P173L	320 (90)
	1	Gag D180N	2700 (360)
	1	Gag T218A	3100 (350)
	1	Gag T218I	2700 (320)
	1	Gag E287T	2700 (300)
**E**	1	WT	1900 (300)
	38	WT	1.2 (0.3)
	38	Gag T55A Q351L	22 (3.9)
	38	Gag K250E	14 (3.1)
**F**	1	WT	1000 (270)
	38	WT	1.5 (0.4)

	1	Gag T55A Q351L	1000 (170)
	1	Gag K250E	5.6 (1.3)
**G**	1	WT	1900 (370)
	38	WT	1 (0.2)
	38	Gag V336I	13 (2.9)
	38	Gag Q350R S395L	18 (2.7)
	38	Gag M174I R362G	51 (17)
	38	Gag I189V S411G	30 (8.8)
**H**	1	WT	2200 (290)
	38	WT	0.5 (0.1)
	1	Gag V336I	3200 (370)
	1	Gag Q350R S395L	1800 (350)
	1	Gag M174I R362G	1900 (150)
	1	Gag I189V S411G	2900 (520)
**I**	1	WT	1400 (200)
	38	WT	1.8 (0.3)
	1	RT D518G/V519A	6.1 (1.1)

	38	RT D518G/V519A	20 (2.9)
I	1	WT	2400 (330)
	38	WT	1 (0.2)
	1	I201T/A273V	190 (19)
	38	I201T/A273V	85 (36)
