# Supplementary material for: The Ty1 Retrotransposon Restriction Factor p22 Targets Gag
Source: PLoS Genet. 2015 Oct 9;11(10):e1005571. doi: 10.1371/journal.pgen.1005571 (PMC4599808; doi:10.1371/journal.pgen.1005571)
Supplement: S5 Table — A Ty1-less S. paradoxus strain with Ty2-917his3-AI (DG3664) was transformed with the following multi-copy plasmids (2μ): (A) empty (pRS416), pGTy2-917 (pGTy2-917) or pTy2-917 (pJC384) (B) empty (pYES2) or p22 (pBDG1565). Cells were grown at 22°C for two days in SC media containing glucose (A) or galactose (B). Numbers represent Ty2his3-AI mobility events per cell and standard deviations are provided in parentheses. Mobility assays were repeated at least three times and representative results are shown. (PDF) [file pgen.1005571.s005.pdf]

S5 Table. Ty2-917*his3-AI* mobility.

	Vector (multicopy, 2μ)	Ty2-917*his3-AI* mobility x 10^{-6} (SD)	Fold change
**A**	empty	6.5 (1.0)	1
	pGTy2-917	5.5 (1.6)	↓1.2
	Ty2-917	9.5 (1.5)	↑1.5
**B**	empty	9.0 (2.7)	1
	p22	4.9 (2.7)	↓1.8
