# Supplementary material for: The Ty1 Retrotransposon Restriction Factor p22 Targets Gag
Source: PLoS Genet. 2015 Oct 9;11(10):e1005571. doi: 10.1371/journal.pgen.1005571 (PMC4599808; doi:10.1371/journal.pgen.1005571)
Supplement: S6 Table — A Ty1-less S. paradoxus with a single Ty1his3-AI (DG2196) was transformed with empty vector pGAL (pBDG101), wild type pGPOLΔ (pBDG1130), pGPOLΔd1 (pBDG1586), or pYES2-p45 (pBDG1375). pGPOLΔ plasmids are deleted for most of POL, produce p22 and confer CNC [26]. The d1 deletion in PR/p4 has been characterized extensively and alters a PR-specific activity involved in reverse transcription and not proteolysis [54, 64]. Note that we are testing for a p22-specific role of PR/p4 sequence, as wild type PR is not produced by pGPOLΔ plasmids due to truncation of POL. The GAG ORF of pYES2-p45 ends at the mature C-terminus of Gag, and thus contains a complete deletion of p4 sequence. Cells were grown in glucose and numbers represent Ty1his3-AI mobility events per cell. Standard deviations are provided in parentheses. (PDF) [file pgen.1005571.s006.pdf]

S6 Table. PR/p4 is not required for CNC.

Vector (multicopy, 2μ)	Ty1*his3-AI* mobility x 10^{-6} (SD)	Fold decrease
empty	490 (140)	1
pGPOLΔ	3.3 (1.5)	150
pGPOLΔ*d1*	3.6 (0.5)	140
pYES2-p45	3.0 (1.7)	163
