# Supplementary figures and images for: The Ty1 Retrotransposon Restriction Factor p22 Targets Gag
Source: PLoS Genet. 2015 Oct 9;11(10):e1005571. doi: 10.1371/journal.pgen.1005571 (PMC4599808; doi:10.1371/journal.pgen.1005571)

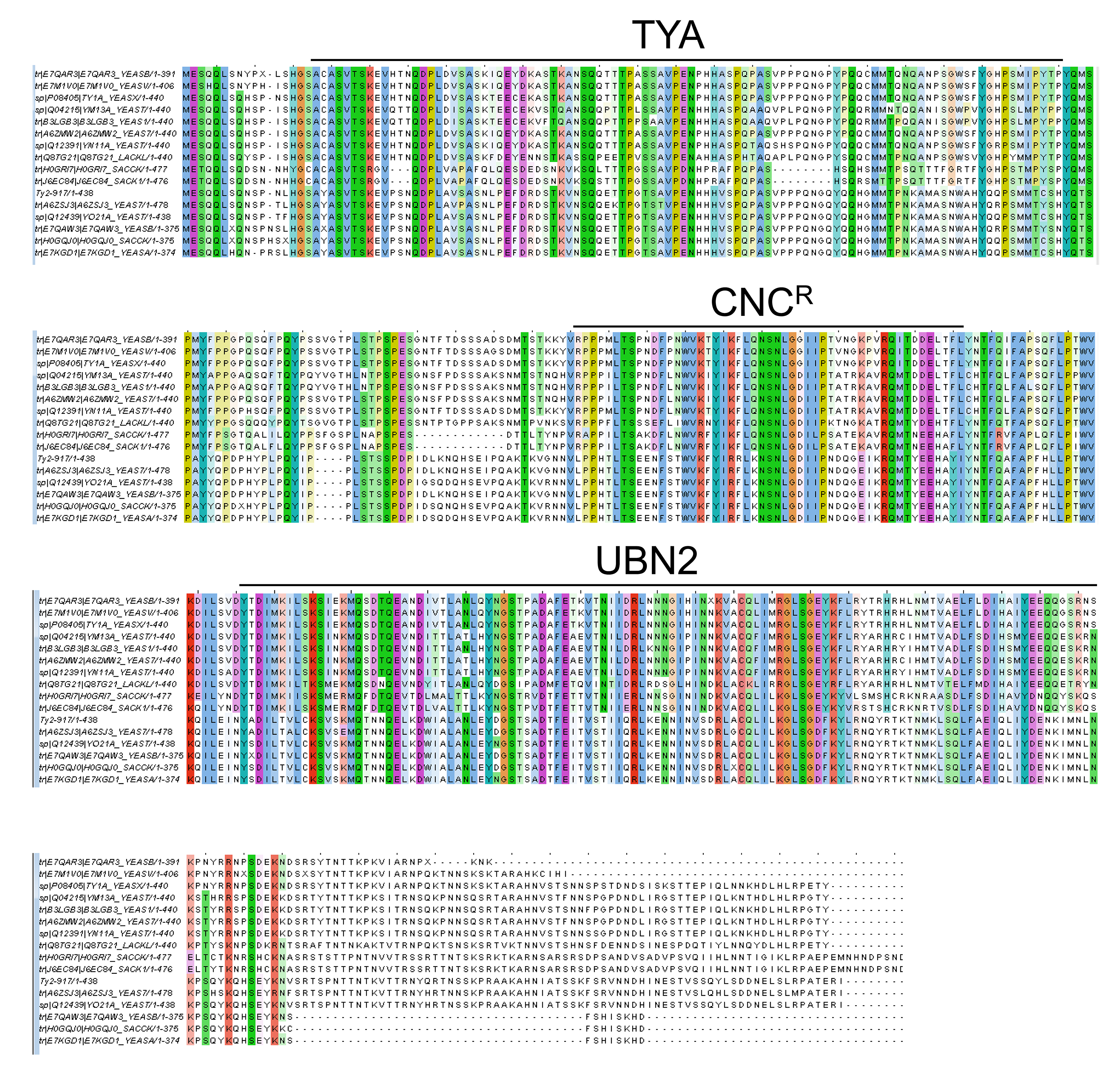

Supplement: S1 Fig — The alignment was generated with ClustalW and visualized with Jalview using the ClustalX color scheme. Uniprot annotations are included to the left and the following domains, which are described in the main text, are labeled: TYA (PF01021), CNCR, and UBN2 (PF14223). Ty1-H3 (P08405) and Ty2-917 were included, however, these elements were isolated as spontaneous retrotransposition events and are unique from known genomic elements [58, 59]. (TIF) [file pgen.1005571.s007.tif]

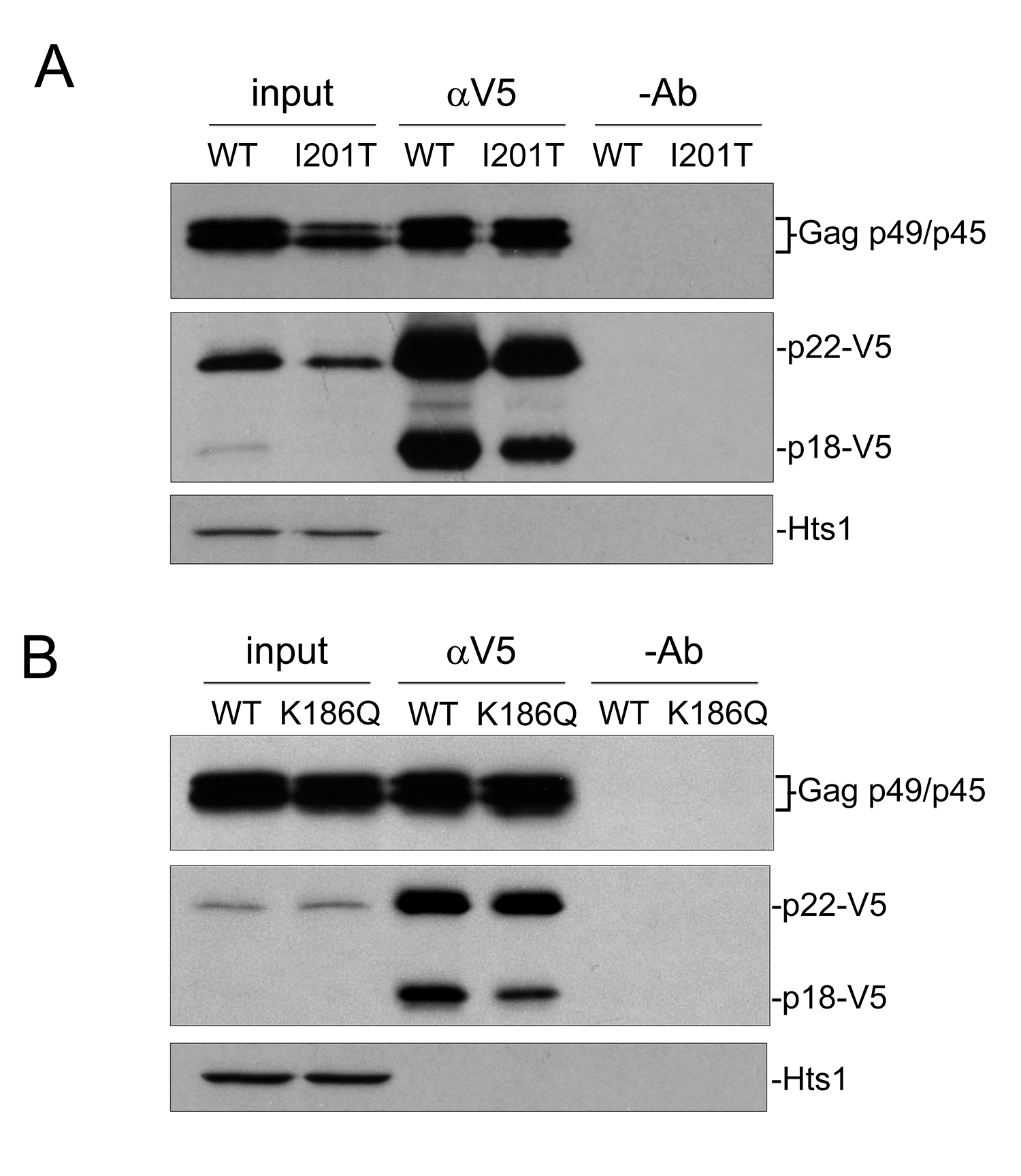

Supplement: S2 Fig — Protein extracts (input) from Ty1-less S. paradoxus strains (DG3508) co-expressing WT (pBDG1534) or pGTy1his3-AI-I201T (A) or pGTy1his3-AI-K186Q (B) and p22-V5 (pBJM93) were incubated with Protein A/G Agarose beads crosslinked to V5 antibody for 2 hours at 4°C. After washing, bound proteins were eluted and immunoblotted with p18 and V5 antibodies. Beads not crosslinked to V5 antibody and Hts1 were used as controls. (TIF) [file pgen.1005571.s008.tif]

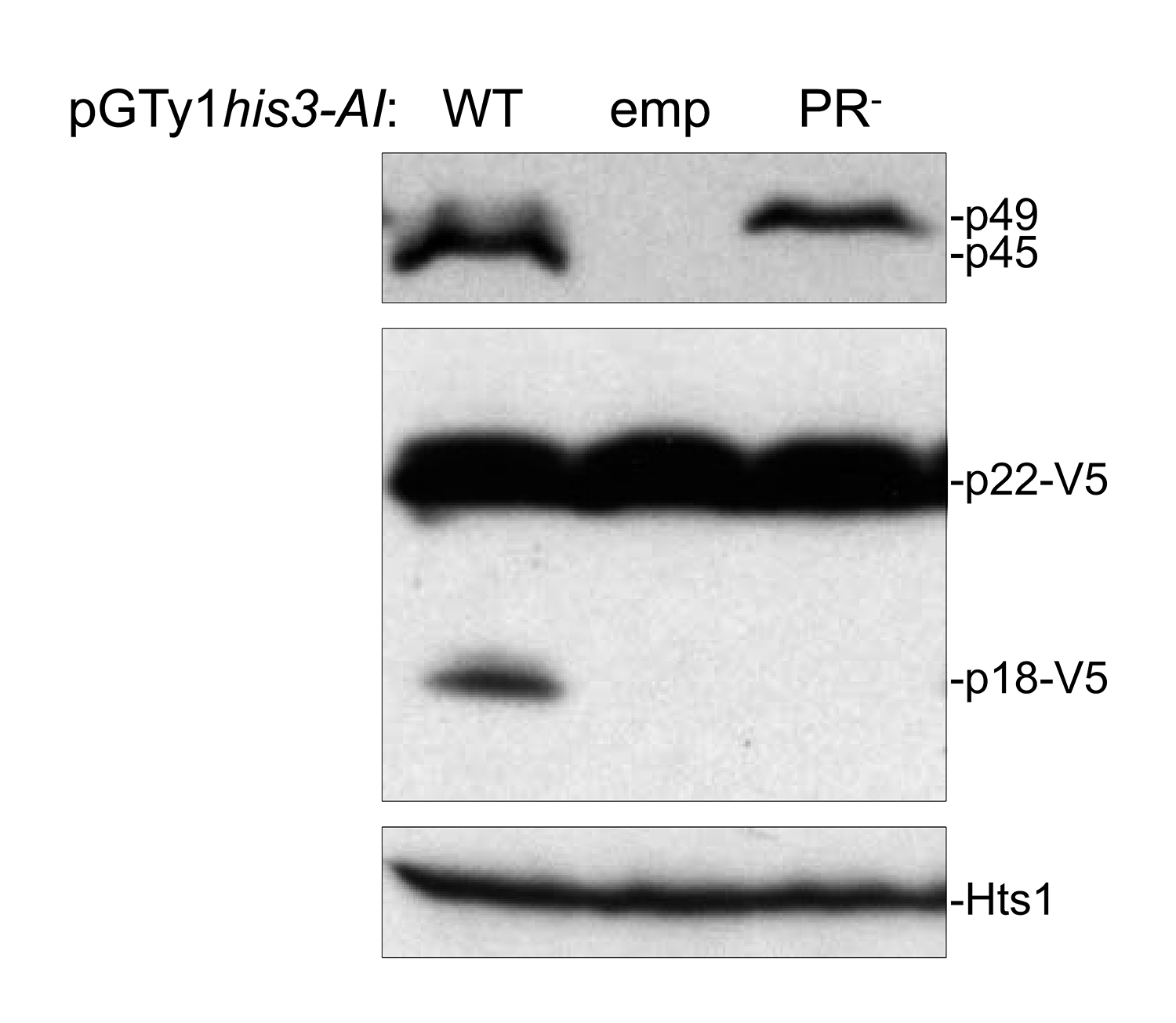

Supplement: S3 Fig — S. paradoxus (DG3508) expressing p22-V5 (pBJM93) was transformed with empty vector (pRS414), wild type (pBDG1534), or PR-defective (PR-) pGTy1his3-AI (pBDG1606). TCA-precipitated extracts were immunoblotted with p18, V5, and Hts1 antibodies. PR was inactivated via a SacI linker insertion at the BglII site in Ty1-H3 [47]. (TIF) [file pgen.1005571.s009.tif]
